# Steering the reaction pathway of methane-to-oxygenates with zero CO_2_ production via photo–thermal catalysis

**DOI:** 10.1093/nsr/nwag472

**Published:** 2026-08-05

**Authors:** Xia Li, Junzhi Wang, Fengxia Wang, Yajie Fu, Peng Wang, Wei Lin, Yu Tang, Lizhi Wu, Li Tan

**Affiliations:** State Key Laboratory of Green and Efficient Development of Phosphorus Resources, College of Chemistry, School of Future Technology, Fuzhou University, Fuzhou 350108, China; State Key Laboratory of Green and Efficient Development of Phosphorus Resources, College of Chemistry, School of Future Technology, Fuzhou University, Fuzhou 350108, China; State Key Laboratory of Green and Efficient Development of Phosphorus Resources, College of Chemistry, School of Future Technology, Fuzhou University, Fuzhou 350108, China; State Key Laboratory of Green and Efficient Development of Phosphorus Resources, College of Chemistry, School of Future Technology, Fuzhou University, Fuzhou 350108, China; State Key Laboratory of Green and Efficient Development of Phosphorus Resources, College of Chemistry, School of Future Technology, Fuzhou University, Fuzhou 350108, China; State Key Laboratory of Green and Efficient Development of Phosphorus Resources, College of Chemistry, School of Future Technology, Fuzhou University, Fuzhou 350108, China; State Key Laboratory of Green and Efficient Development of Phosphorus Resources, College of Chemistry, School of Future Technology, Fuzhou University, Fuzhou 350108, China; State Key Laboratory of Green and Efficient Development of Phosphorus Resources, College of Chemistry, School of Future Technology, Fuzhou University, Fuzhou 350108, China; State Key Laboratory of Green and Efficient Development of Phosphorus Resources, College of Chemistry, School of Future Technology, Fuzhou University, Fuzhou 350108, China

**Keywords:** selective CH_4_ conversion, photo–thermal synergistic catalysis, methanol, formaldehyde, shielding overoxidation pathway

## Abstract

The direct conversion of CH_4_ into liquid oxygenates via heterogeneous catalysis is highly challenging due to the facile overoxidation of target products. Herein, we present a potential Ag-modified In_2_O_3_ photo–thermal catalyst that enables the selective oxidation of CH_4_ to CH_3_OH and HCHO using molecular O_2_ under mild conditions (150°C, Xe lamp irradiation). The optimized Ag-3/In_2_O_3_ catalyst exhibits remarkable performance, achieving 100% selectivity toward liquid oxygenates with complete suppression of CO_2_ formation, and delivers a high production rate of 6544 μmol · g^−1^ · h^−1^, 2.6 times greater than that under pure photocatalysis at 25°C. Mechanistic studies reveal that photogenerated holes facilitate C–H bond activation to form ·CH_3_ radicals, while O_2_ is selectively reduced at electron-rich Ag sites to generate ·OH species. Meanwhile, it is demonstrated that excessive ·OH generation on the Ag-10/In_2_O_3_ contrast catalyst promotes HCHO overoxidation to CO_2_, highlighting the importance of ·OH regulation in determining product selectivity over the Ag-3/In_2_O_3_ catalyst. Moreover, combined with experiments and DFT calculations, the results demonstrate that In_2_O_3_ with a suitable band structure exhibits weak adsorption affinity toward CH_3_OH and HCHO, while also providing an in-depth understanding of the zero CO_2_ emissions in this catalytic process. The work illustrates that the strategic coupling of thermal and photocatalytic pathways enhances charge-carrier utilization and promotes selective radical chemistry for efficient CH_4_ valorization.

## Teaser

An Ag-modified In2O3 photo–thermal catalyst converts CH4 into liquid oxygenates with 100% selectivity and a high yield of 6544 μmol · g − 1 · h − 1 at 150°C under Xe lamp irradiation.

## INTRODUCTION

The conversion of natural gas into transportable fuels and high-value chemicals is of increasing strategic importance, spurred by emerging industrial trends [1]. These include the growing global demand for methanol and its derivatives, a trend further reinforced by the expanding use of methanol fuel cells and the commercialization of methanol to olefins (MTO) technology [2,3]. As the main constituent of natural gas, methane (CH_4_) is considered a key energy carrier owing to its abundant reserves, high energy density, and relatively low carbon emissions [4–7]. Nevertheless, the efficient conversion of CH_4_ to methanol (CH_3_OH) and formaldehyde (HCHO) remains a major scientific challenge. This stems from two inherent difficulties: first, the CH_4_ molecule possesses a strong C–H bond (439 kJ ·  mol^−1^), which demands elevated temperatures for activation [8–10]; second, the target oxygenates CH_3_OH and HCHO have significantly lower oxidation potentials than CH_4_ itself [11–13], making them prone to overoxidation to CO_2_. This inevitable overoxidation severely limits product selectivity. Consequently, the central challenges in this transformation are to achieve high activity, high selectivity, and efficient carbon atom utilization.

Thermocatalytic approaches for CH_4_ activation typically require harsh conditions, including high reaction temperature (>500°C), strong oxidants such as H_2_O_2_, or the introduction of gases (CO/H_2_) to *in situ* generate H_2_O_2_ from O_2_ [14–19]. Although this approach can enhance CH_4_ conversion, it suffers from poor product selectivity due to overoxidation. The current limitations have motivated the search for more efficient reaction technologies. Recently, photocatalytic CH_4_ oxidation has emerged as a promising alternative [20,21], utilizing photon energy to overcome activation barriers and drive selective transformations under mild conditions, thereby circumventing the thermodynamic constraints of conventional thermocatalytic processes. Typically, semiconductors (e.g. ZnO, TiO_2_, and WO_3_) have been demonstrated to facilitate photocatalytic CH_4_ oxidation using O_2_ as the terminal oxidant [22–24], as well as highlighting the considerable potential of noble metal-modified metal oxides. However, the overall catalytic performance of photocatalytic systems remains constrained, largely due to the rapid recombination of photogenerated electrons and holes at room temperature, which leads to sluggish reaction kinetics.

To tackle these issues, photo–thermal catalysis has emerged as a promising approach [25]. Introducing additional thermal energy can not only accelerate the desorption of intermediate products to prevent overoxidation but also promote the separation of photogenerated electrons and holes, leading to improved CH_4_ conversion efficiency. Lately, Tang *et al*. [26] reported a photon–phonon-driven cascade reaction over a Ru single-atom catalyst (Ru_SA_-ZnO), converting CH_4_ to HCHO at 150°C with a productivity of 40 150 μmol · g^−1^ · h^−1^ and a selectivity of 90.4%. Nevertheless, a substantial amount of CO_2_ (3800 μmol · g^−1^ · h^−1^) was still generated, indicating significant carbon loss. In another study, Wu *et al*. [27] developed a continuous-flow gas–solid photo–thermal system using an Ag single-atom-modified ZnO catalyst (Ag_1_-ZnO), achieving an HCHO productivity of 29 450 μmol · g^−1^ · h^−1^ with 71.2% selectivity. Although photo–thermal catalysis markedly enhances product yield, the required thermal input inevitably enhances the undesired overoxidation of CH_4_ to CO_2_, which remains a persistent bottleneck that compromises carbon efficiency and overall process selectivity.

Previous research suggests that modulating the reaction pathway can be an effective strategy for improving catalytic selectivity in photo–thermal systems. Overoxidation is mainly driven by hydroxyl radicals (·OH) and/or photogenerated holes [28,29], which are typically formed through two primary routes in photocatalysis: (i) oxidation of H_2_O by holes, and (ii) activation of O_2_ by electrons. Consequently, suppressing the ·OH radical generation has emerged as a key direction for selectivity control. One approach involves constructing heterojunctions to facilitate the transfer or trapping of holes, thereby lowering their oxidizing ability toward H_2_O and reducing the ·OH radical formation. For instance, Ye *et al*. [8] developed a dual-cocatalyst system by co-loading metal nanoparticles (Au, Pd, Ag, Pt) and cobalt oxide (CoO*_x_*) nanoclusters on a TiO_2_ carrier, achieving highly selective photocatalytic oxidation of CH_4_ (the selectivity of methyl hydroperoxide (CH_3_OOH) and CH_3_OH is 95%, with a space–time yield (STY) of 2540 μmol · g^−1^ · h^−1^) at room temperature. Alternatively, narrow-bandgap semiconductors with suitably positioned valence bands can intrinsically suppress the ·OH radical production. Cubic In_2_O_3_ (*E*_g_ = 2.6  eV), for example, exhibits a maximum valence band below the H_2_O oxidation potential, which effectively limits the ·OH radical generation. Tang *et al*. [30] reported an Au*_NPs_*/In_2_O_3_ catalyst that delivered a STY of 2069 μmol · g^−1^ · h^−1^ for liquid products with 91.04% selectivity under ambient conditions. Similarly, Li *et al*. [31] prepared a single-atom Pt-modified ZnO (Pt_1_/ZnO) catalyst, enabling photocatalytic CH_4_ oxidation to CH_3_OH and HCHO with a STY of 4600 μmol · g^−1^ · h^−1^ and 95% selectivity. In summary, the rational design of noble metal-modified semiconductor catalysts to regulate the ·OH radical formation offers a promising route toward high-selectivity photo–thermal CH_4_ conversion.

Herein, we report an efficient photo–thermal synergistic catalysis for the direct conversion of CH_4_ into valuable oxygenates. For this purpose, a rationally designed Ag nanoparticle-modified In_2_O_3_ catalyst (denoted as Ag-*x*/In_2_O_3_) was developed. Notably, Ag offers a more cost-effective alternative to noble metals such as Pt, Au, or Ru [32–36], while In_2_O_3_ with a suitable band structure enables weaker adsorption affinity toward CH_3_OH and HCHO, compared to common photocatalytic semiconductors (e.g. ZnO, TiO_2_-A, and P25). Under Xe lamp irradiation at 150°C, the Ag-3/In_2_O_3_ catalyst achieves a productivity of 6544 μmol · g^−1^ · h^−1^ with 100% selectivity toward liquid oxygenates, presenting a 2.6–fold enhancement compared to its performance at 25°C. *In situ* electron paramagnetic resonance (EPR) and photoluminescence (PL) studies reveal that photon energy helps overcome thermodynamic barriers and promotes CH_4_ activation under mild conditions, while thermal energy accelerates the overall reaction kinetics. As a result, this work demonstrates an efficient and selective route for CH_4_ oxidation to liquid oxygenates with complete suppression of CO_2_ formation under mild photo–thermal conditions.

## RESULTS AND DISCUSSION

### Structural analysis of Ag-*x*/In_2_O_3_ catalysts

The synthesis of Ag-modified In_2_O_3_ (Ag-*x*/In_2_O_3_) catalysts via a NaBH_4_ reduction method [37] is schematically illustrated in Fig. 1a. The actual Ag loadings, determined by inductively coupled plasma optical emission spectrometry (ICP–OES), are summarized in Table S1. Aberration-corrected high-angle annular dark-field scanning transmission electron microscopy (AC-HAADF-STEM) images of the representative Ag-3/In_2_O_3_ and Ag-10/In_2_O_3_ samples clearly reveal that surface Ag species with different atomic mass contrasts are confined on the In_2_O_3_ matrix. Energy–dispersive X-ray spectroscopy (EDS) elemental mapping confirms the homogeneous distribution of Ag nanoparticles in the Ag-3/In_2_O_3_ sample across the In_2_O_3_ support. However, with the increase in the loading amounts, Ag particles on the Ag-10/In_2_O_3_ sample exhibit obvious agglomeration (Fig. 1b–e). Furthermore, the definite characteristic signals attributed to Ag species are demonstrated via the EDS spectra (Fig. S1). X-ray diffraction (XRD) patterns (Fig. 1f) show that an additional diffraction peak emerges at 2*θ*=38.1°, indexed to metallic Ag nanoparticles (PDF#04–0783) for both Ag-3/In_2_O_3_ and Ag-10/In_2_O_3_. In contrast, no distinct Ag crystalline phase is detected in the samples with lower Ag loading, indicating a highly dispersed state of the Ag species. UV-visible diffuse reflectance spectroscopy (UV-vis DRS) (Fig. 1g) reveals a pronounced absorption band around 420 nm for the Ag-*x*/In_2_O_3_ series, characteristic of the surface plasmon resonance arising from metallic Ag nanoparticles [22], consistent with the structural analysis of the catalysts described above.

**Figure 1.**
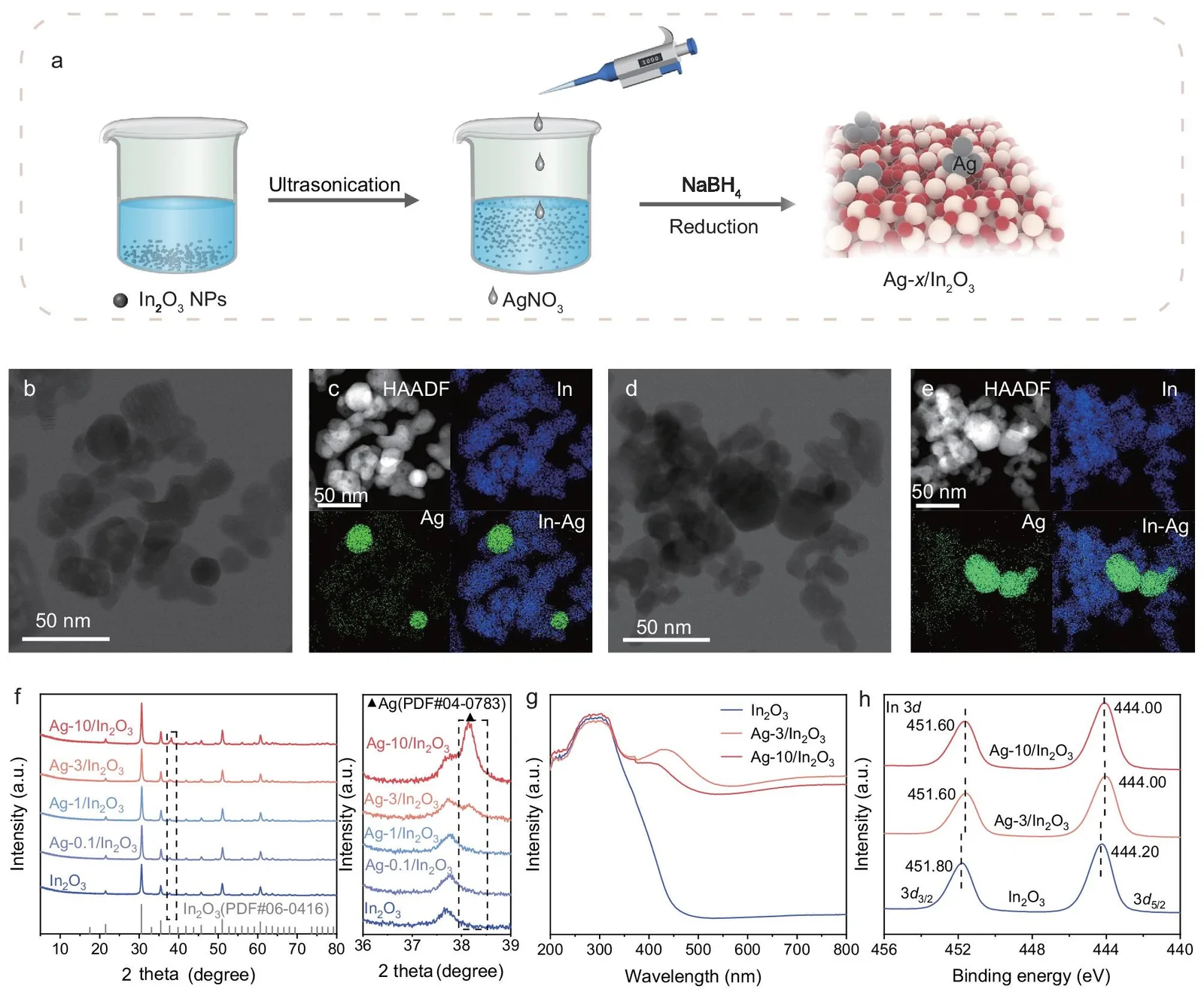
The catalyst preparation and structural characterizations. (a) Schematic illustration of the synthetic procedure of Ag-*x*/In_2_O_3_. AC-HAADF-STEM images of (b, c) Ag-3/In_2_O_3_ and (d, e) Ag-10/In_2_O_3_ with corresponding EDX elemental mapping images. (f) XRD patterns of the series of catalysts. (g) UV-vis spectra of different catalysts. (h) XPS spectra of different catalysts.

X-ray photoelectron spectroscopy (XPS) was carried out to explore the local structure of surface Ag species in catalysts. The Ag 3*d* spectra confirm that the loaded Ag species in both Ag-3/In_2_O_3_ and Ag-10/In_2_O_3_ catalysts exist predominantly in the metallic state (Fig. S2). However, a lower shift of binding energy appeared at Ag-3/In_2_O_3_ and Ag-10/In_2_O_3_ catalysts in the 3*d* spectra (Fig. 1h), indicating electron transfer from the Ag nanoparticles to the In_2_O_3_ support [38,39]. This interfacial electron redistribution is beneficial for the directional migration and separation of photogenerated charge carriers. Furthermore, the quantitative analysis of surface O species reveals an increase in the relative concentration of oxygen defects after Ag loading (Fig. S3). As reported, the enhancement is ascribed to the use of NaBH_4_ as a strong reducing agent, which not only reduces Ag^+^ to metallic Ag but also simultaneously reduces a portion of the surface oxygen atoms on the In_2_O_3_ support, thereby generating additional oxygen vacancies [40].

### The photo–thermal synergistic catalytic performance of CH_4_ oxidation

The photo–thermal catalytic performance in CH_4_ oxidation was evaluated in a batch reactor (Fig. S4). Possible liquid products were quantified by ^1^H nuclear magnetic resonance (NMR) and a colorimetric method, and the gas products were measured by gas chromatography (GC) (Figs S5–S7). Firstly, the effect of catalyst supports on the liquid oxygenates selectivity was investigated. The semiconductor supports exhibit distinct behaviors in governing their oxidation process. It is noted that In_2_O_3_ effectively suppressed the overoxidation of liquid oxygenates, while the use of ZnO, TiO_2_-A, and P25 tended to promote complete oxidation and, as a consequence, higher CO_2_ yields (Fig. 2a). This divergence comes from inherent support band properties, with ZnO, TiO_2_-A, and P25 exhibiting stronger adsorption of reaction intermediates (CH_3_OH and HCHO), driving their complete oxidation to CO_2_.

**Figure 2.**
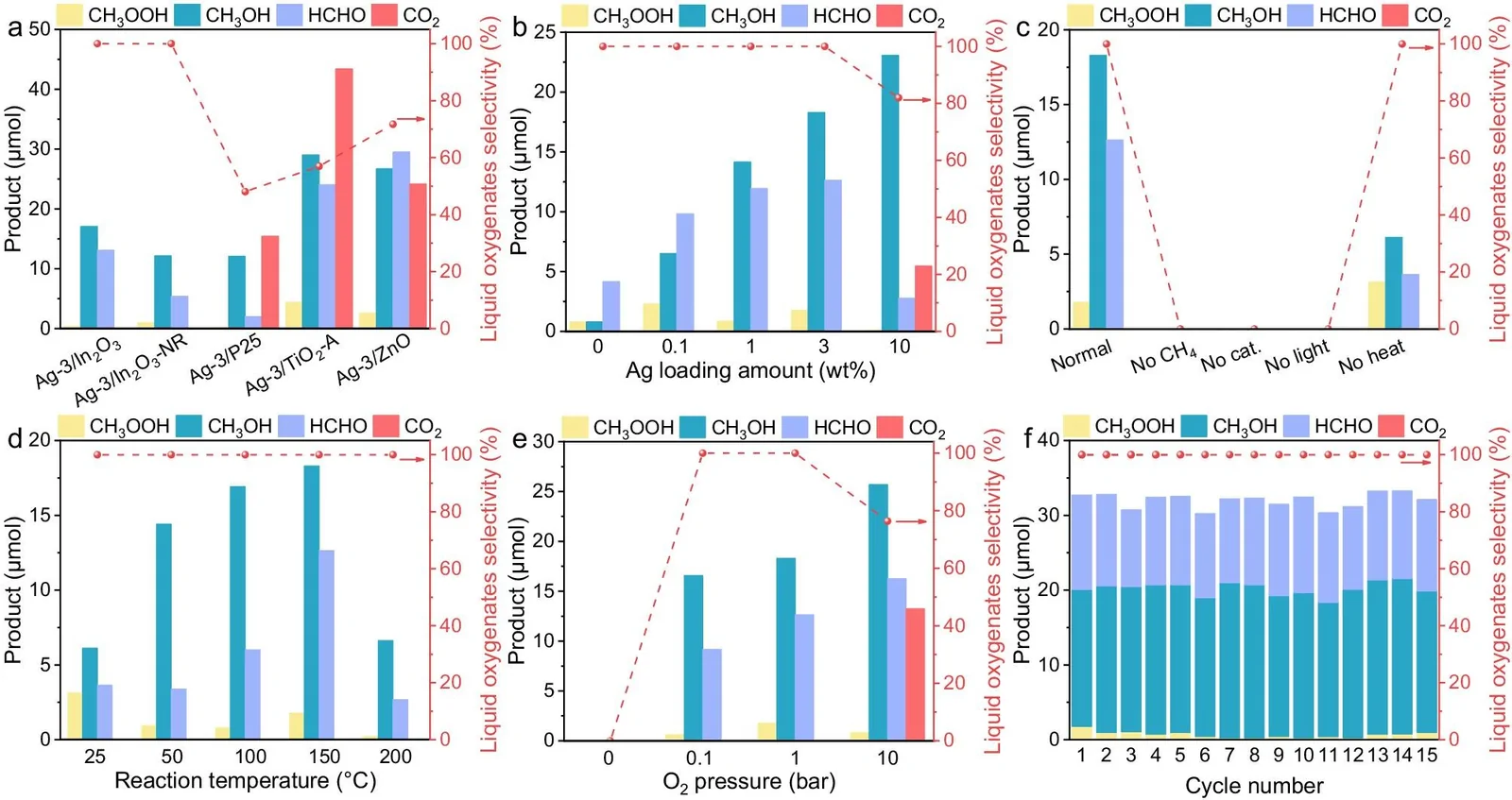
The catalytic performance evaluation of the photo–thermal CH_4_ conversion reaction. (a) The yield and selectivity of liquid oxygenates over various catalysts. (b) The yield and selectivity of liquid oxygenates over Ag-*x*/In_2_O_3_. The yield and selectivity of liquid oxygenates over Ag-3/In_2_O_3_: (c) control experiments, (d) different reaction temperatures, (e) different O_2_ pressures, and (f) cycling tests of the same Ag-3/In_2_O_3_ catalyst. Typical reaction conditions: Xenon lamp (320–780 nm), 5 mg catalyst, 39 bar CH_4_, 1 bar O_2_, 150°C, 1 h, 20 mL H_2_O. Other condition variables are according to the parameters shown in the figures.

As illustrated in Fig. 2b and Table S2, the pure In_2_O_3_ support produces only 5.77 μmol of liquid oxygenates with negligible CO_2_ formation. HCHO is the dominant liquid product (4.17 μmol, 72.27% selectivity), along with minor amounts of CH_3_OOH (0.80 μmol) and CH_3_OH (0.80 μmol). Ag, as an excellent electron trapper, can significantly promote photo–thermal catalytic CH_4_ conversion. Thus, the selectivity of HCHO has decreased, and CH_3_OH selectivity is enhanced with increasing Ag loading amounts. Specifically, for the Ag-3/In_2_O_3_ catalyst, the total yield of liquid oxygenates increases significantly to 32.72 μmol (equivalent to 6544 μmol · g^−1^ · h^−1^) with 100% selectivity. CH_3_OH constitutes the dominant product, yielding 18.30 μmol at 55.92% selectivity, while HCHO production also increases substantially to 12.64 μmol, with a corresponding selectivity of 38.63%. Moreover, the Ag-3/In_2_O_3_ catalyst embodies the optimized catalytic performance that surpasses most reported catalysts (Table S3). Further investigation of the increased amounts of Ag loading on catalytic performance, the Ag-10/In_2_O_3_ catalyst reflects an increased CH_3_OH yield (23.08 μmol), but the HCHO production decreases significantly to 5.47 μmol, and the emergence of CO_2_ as a byproduct decreases the liquid oxygenates selectivity to 82%. Additionally, the contribution of particle size effects to the reaction performance was ruled out (Fig. S8). The product selectivity distribution influenced by varied Ag loading as a function implies the feasibility of a modulated reaction pathway.

The roles of light and heat were verified by setting up control experiments using the optimal catalyst (Ag-3/In_2_O_3_) (Fig. 2c). In the absence of thermal input, the yield of liquid oxygenates decreases by approximately 20 μmol compared to that at 150°C conditions. However, no products are detected over Ag-3/In_2_O_3_ at 150°C under dark conditions. These results clearly demonstrate that this reaction is activated via photon energy and promotes catalytic efficiency through heat input. Control experiments further confirmed that no products were detected in the absence of either catalyst or CH_4_ feed, unequivocally indicating that all products originate from the photo–thermal catalytic conversion of CH_4_. The influence of reaction temperatures was further examined on the Ag-3/In_2_O_3_ catalyst. As shown in Fig. 2d, the yield of liquid oxygenates exhibits a volcanic trend with increasing temperature, reaching a maximum at 150°C, 2.6 times higher than that at 25°C. This trend is consistent with *in situ* PL analysis (Fig. S9), where the PL intensity progressively decreases as temperature rises, suggesting that heating markedly facilitates the separation of photogenerated carriers and thereby enhances the catalytic activity.

The partial pressure of O_2_ for the selective formation of liquid oxygenates was investigated over Ag-3/In_2_O_3_ (Fig. 2e). No oxygenates are produced in the absence of O_2_, providing evidence that O_2_ plays an indispensable role in the formation of oxygenates. With the partial pressure of O_2_ rising to 1 bar, the yield of liquid oxygenates significantly increased to 32.72 μmol with a selectivity of 100%. However, increasing the O_2_ pressure to 10 bar leads to an additional rise in liquid oxygenates yield, accompanied by enhanced overoxidation to CO_2_. Reaction time is another key factor in the photo–thermal catalytic oxidation of CH_4_ (Fig. S10). As the reaction time increases, the yield of liquid oxygenates gradually rises.

Cyclic experiments were conducted to evaluate the stability of Ag-3/In_2_O_3_. As shown in Fig. 2f, the yield and selectivity of liquid oxygenates remain almost identical after 15 rounds of reaction cycles. XRD (Fig. S11), TG (Fig. S12), AC-HAADF-STEM (Fig. S13), and XPS (Fig. S14) characterizations reveal no significant changes in the crystalline phase and electronic structure of Ag-3/In_2_O_3_ before and after the reaction. Furthermore, considering the possible leaching of Ag species, the filtrates collected after the reaction cycles of the 5th, 10th, and 15th were analyzed by ICP–OES. The results indicate that the Ag content in the different filtrates is almost negligible, suggesting that there is no Ag leaching during the reaction process. These results provide evidence for the excellent stability of Ag-3/In_2_O_3_.

### The selective activation pathway of methane and oxygen molecules

The reaction mechanism of the photo–thermal synergistic catalysis is further systematically investigated. This catalytic process primarily involves three fundamental steps: light absorption, charge separation, and surface reaction [41]. The light absorption properties of these samples were characterized by UV-vis DRS (Fig. 1g). The introduced Ag extends the light response range from the UV region to the visible region (λ=400–800 nm) [42,43]. To elucidate the charge separation dynamics, comprehensive photoelectrochemical and steady-state PL characterizations were performed [44,45]. As demonstrated by photocurrent response, electrochemical impedance spectroscopy (EIS), and PL spectra analyses (Fig. S15 and Fig. 3a), the incorporation of Ag significantly improves the photogenerated charge separation efficiency.

**Figure 3.**
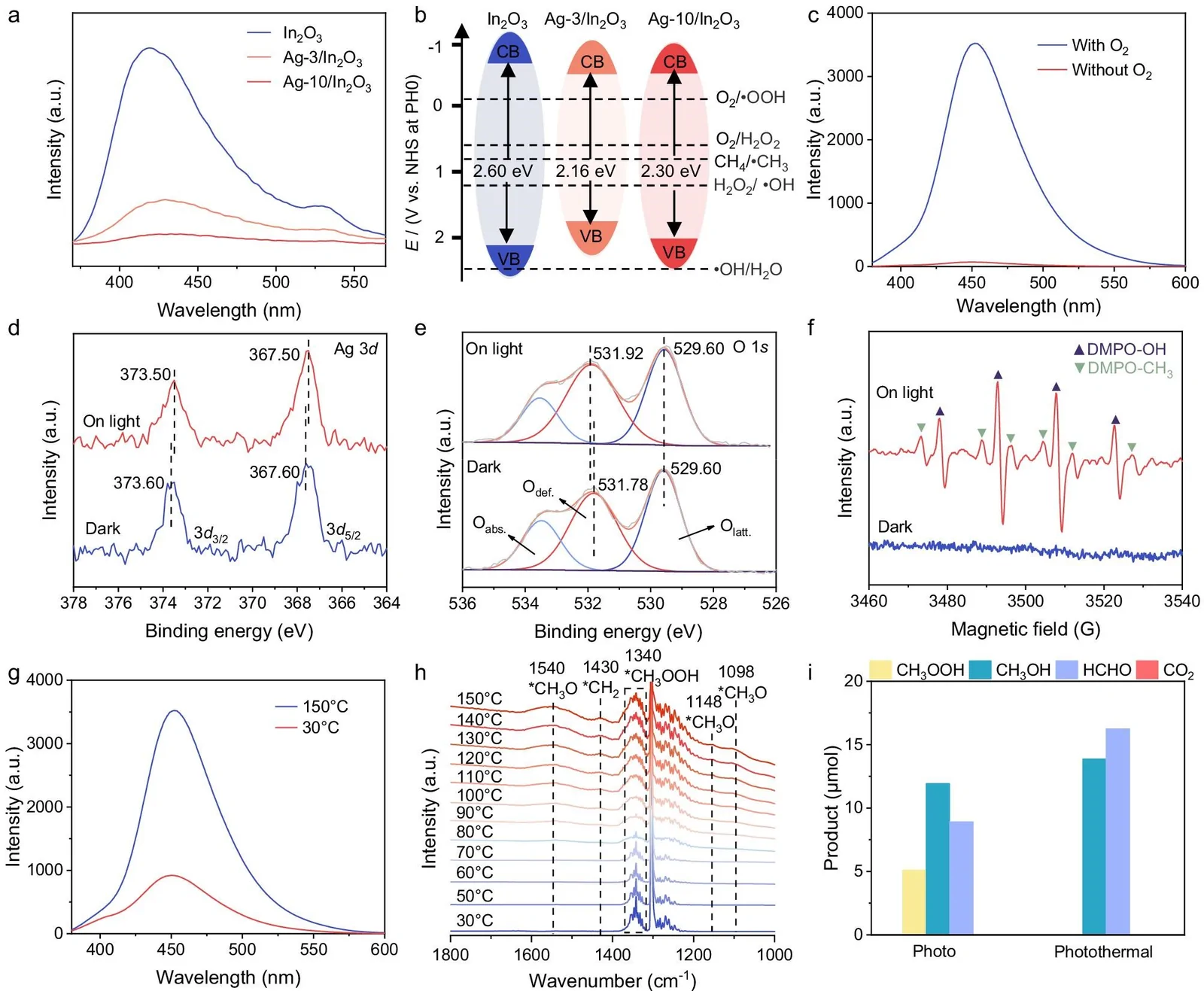
Reaction mechanism study of the photo–thermal catalytic oxidation of CH_4_ with O_2_ at the molecular level. (a) PL spectra of different catalysts. (b) Band structures of different catalysts with the redox potential of typical radicals. (c) PL spectra of 7-HC over Ag-3/In_2_O_3_ under O_2_ or Ar atmosphere. (d and e) XPS spectra under dark or light conditions of Ag-3/In_2_O_3_. (f) *In situ* EPR spectra under dark or light conditions of Ag-3/In_2_O_3_. (g) PL spectra of 7-HC over Ag-3/In_2_O_3_ at 30°C and 150°C. (h) *In situ* DRIFTS spectra of photo–thermal catalytic CH_4_ conversion over Ag-3/In_2_O_3_. (i) Photocatalytic CH_4_ conversion over Ag-3/In_2_O_3_ first at 25°C under light irradiation for 2 h and then kept at 150°C in the dark for 1 h.

After obtaining information about the photogenerated charge kinetics for the catalysts, the activation behaviors of reactants and intermediate species were further examined. The Ag-3/In_2_O_3_ is estimated to have a direct band gap (*E*_g_) of 2.16 ± 0.05 eV through Kubelka–Munk transformation of UV-vis data (Fig. S16a) [46,47]. The conduction band potential (*E*_CB_) is then calculated according to Mott–Schottky plots to be −0.285 ± 0.05 eV vs. NHE (normal hydrogen electrode) (Fig. S16b). Following the relationship between the *E*_CB_ and the valence band (*E*_VB_), i.e. *E*_VB_ = *E*_CB_ − *E*_g_, the *E*_VB_ is deduced to be 1.875 ± 0.05 eV. As illustrated in Fig. 3b, this band alignment creates a unique situation; the *E*_VB_ position of all catalysts lies above the oxidation potential for H_2_O to ·OH radicals (·OH/H_2_O = 2.38 eV), indicating that the photogenerated holes in the valence band cannot oxidize H_2_O into ·OH radicals. In contrast, the *E*_VB_ position of all catalysts is situated below the oxidation potential for CH_4_ to ·CH_3_ radicals (·CH_3_/CH_4_ = 0.83 eV) [38,48], revealing that the photogenerated holes of In_2_O_3_ possess sufficient energy to activate the C–H bond in CH_4_. Consequently, OH radicals must originate exclusively from O_2_ reduction pathways. The rational contrast experiments were designed to testify to our deduction. The coumarin, as a fluorescent probe, is used for the PL experiments to identify ·OH radicals produced under individual O_2_ or Ar atmospheres. After degassing via Ar purging (Fig. 3c), the intense fluorescence signal of 7-hydroxycoumarin (7-HC) disappears on Ag-3/In_2_O_3_, providing direct proof that ·OH radicals derive from O_2_ reduction by photogenerated electrons. In addition, *in situ* EPR measurements under anaerobic and aerobic conditions also corroborated this conclusion (Fig. S17).

The electron transfer mechanism is probed by *in situ* XPS analysis (Fig. 3d, e and Fig. S18). Under light irradiation, the characteristic peaks of Ag 3*d*_5/2_ (367.50 eV) and Ag 3*d*_3/2_ (373.50 eV) exhibit a negative shift of 0.10 eV, while the O 1 *s* feature associated with oxygen–vacancy-related oxygen species displays a positive shift. These observations suggest a well-defined electron transfer pathway: (i) photogenerated electrons (e^−^) are excited from the valence band to the conduction band of In_2_O_3_ and transfer to Ag nanoparticles, driving the O_2_ reduction pathway; (ii) simultaneously, photogenerated holes (h^+^) accumulate at oxygen sites of In_2_O_3_, facilitating the cleavage of the C–H bond in CH_4_ to form ·CH_3_ radicals [49]. Direct evidence for radical formation was presented from *in situ* EPR experiments using 5,5–dimethyl–1–pyrroline *N*–oxide (DMPO) as the capture agent (Fig. 3f). Without illumination, no free radicals are generated in the Ag-3/In_2_O_3_ catalysis system. Meanwhile, the characteristic signals of both ·CH_3_ radicals and ·OH radicals are detected over Ag-3/In_2_O_3_ in a CH_4_ atmosphere under light illumination, which is a clear indication that C–H activation proceeds via a light-driven radical pathway.

To distinguish the underlying activation mechanisms, we introduced an excess of sodium oxalate (Na_2_C_2_O_4_) as an h^+^ scavenger and coumarin as a fluorescent probe for ·OH radicals into the reaction system [23]. The results show that Ag-3/In_2_O_3_ retains its capacity to produce ·OH radicals even after effective h^+^ quenching (Fig. S19a). However, under the same conditions, the production rate of liquid oxygenates in photo–thermal catalytic CH_4_ oxidation decreased by approximately 85% (Fig. S19b), suggesting a dominant role of h^+^ rather than ·OH radicals in activating the first C–H bond of CH_4_.

Having systematically established the role of light, we turned to evaluating the contribution of thermal energy in the photo–thermal catalytic oxidation of CH_4_ over Ag-3/In_2_O_3_. Elevation of the reaction temperature from 25°C to 150°C yields a 2.6-fold enhancement in liquid oxygenates production (from 12.91 to 32.72 μmol) at a constant 100% selectivity (Fig. 2d). To understand this thermal promotion effect, semi-quantitative detection experiments of the ·OH radical concentrations at various temperatures were performed using the coumarin probe method (Fig. 3g). The fluorescence signal of 7-HC at 150°C (intensity of 3522 a.u.) is more intense than that at 30°C (intensity of 920 a.u.), indicating that an elevated temperature promotes ·OH radical formation. This result is consistent with *in situ* PL studies, in which higher temperatures suppress the recombination of photogenerated electrons and holes, thereby promoting electron-driven O_2_ reduction. Together, these exploration experiments of activation mechanisms underscore the essential role of thermal energy in accelerating reaction kinetics and boosting product formation.

Further insights into the input heating were obtained through *in situ* DRIFTS (Fig. 3h). The CH_4_/O_2_ mixed gas was bubbled through deionized water to allow saturated water vapor to enter the reaction cell. After the system had been stabilized for 30 minutes, the reaction temperature was raised from 30°C to 150°C at a rate of 5°C/min, and the infrared spectra during this process were recorded. In the spectral region of 1800–1200 cm^−1^, characteristic absorption bands associated with oxygenated intermediates are identified: the peaks at 1550, 1148, and 1098 cm^−1^ are attributed to the C–O stretching vibrations of surface methoxy (*OCH_3_) species [9,26,35,50–52], while the absorption band at 1430 cm^−1^ is assigned to the C–H deformation vibration of surface methylene (*CH_2_) groups [36,53]. A distinct peak at 1340 cm^−1^ is also identified, characteristic of *CH_3_OOH species [36,54,55]. On the Ag-3/In_2_O_3_ surface, the signals of *OCH_3_ (from adsorbed CH_3_OH) and *CH_2_ (from adsorbed HCHO) intensify with temperature, coupled with the diminishment of the *CH_3_OOH signal, which demonstrates thermally promoted decomposition of the peroxide intermediate. These results agree with experimental observations: thermal input promotes the selective conversion of CH_4_ to liquid oxygenates.

To corroborate the transformation pathway suggested by the *in situ* DRIFTS data, we conducted separate CH_3_OOH decomposition experiments (Fig. 3i). CH_3_OOH is produced by the photocatalytic oxidation of CH_4_ at 30°C and is then subjected to a thermal step at 150°C without light irradiation. The secondary step leads to all CH_3_OOH molecules being transformed to CH_3_OH and HCHO, with HCHO as the primary product (80%). This result provides direct experimental validation for the proposed mechanism in which CH_3_OOH serves as the key precursor to HCHO.

### Controlling the oxidation process and mechanism study

To clarify the pathway of CO_2_ generation, mechanistic studies involving intermediate oxidation and radical trapping experiments were conducted upon the Ag-3/In_2_O_3_ catalysis system. Under the same reaction conditions with 37 μmol of HCHO as feedstock, 14.49 μmol of HCHO remains after the reaction. Upon addition of the hole scavenger Na_2_C_2_O_4_, the residual HCHO amounts to 15.71 μmol. In contrast, introduction of the ·OH radical scavenger salicylic acid substantially increases the remaining HCHO to 34.79 μmol, underscoring the decisive role of ·OH radicals in driving HCHO overoxidation (Fig. 4a). Parallel experiments with CH_3_OH also confirmed this conclusion (Fig. S20).

**Figure 4.**
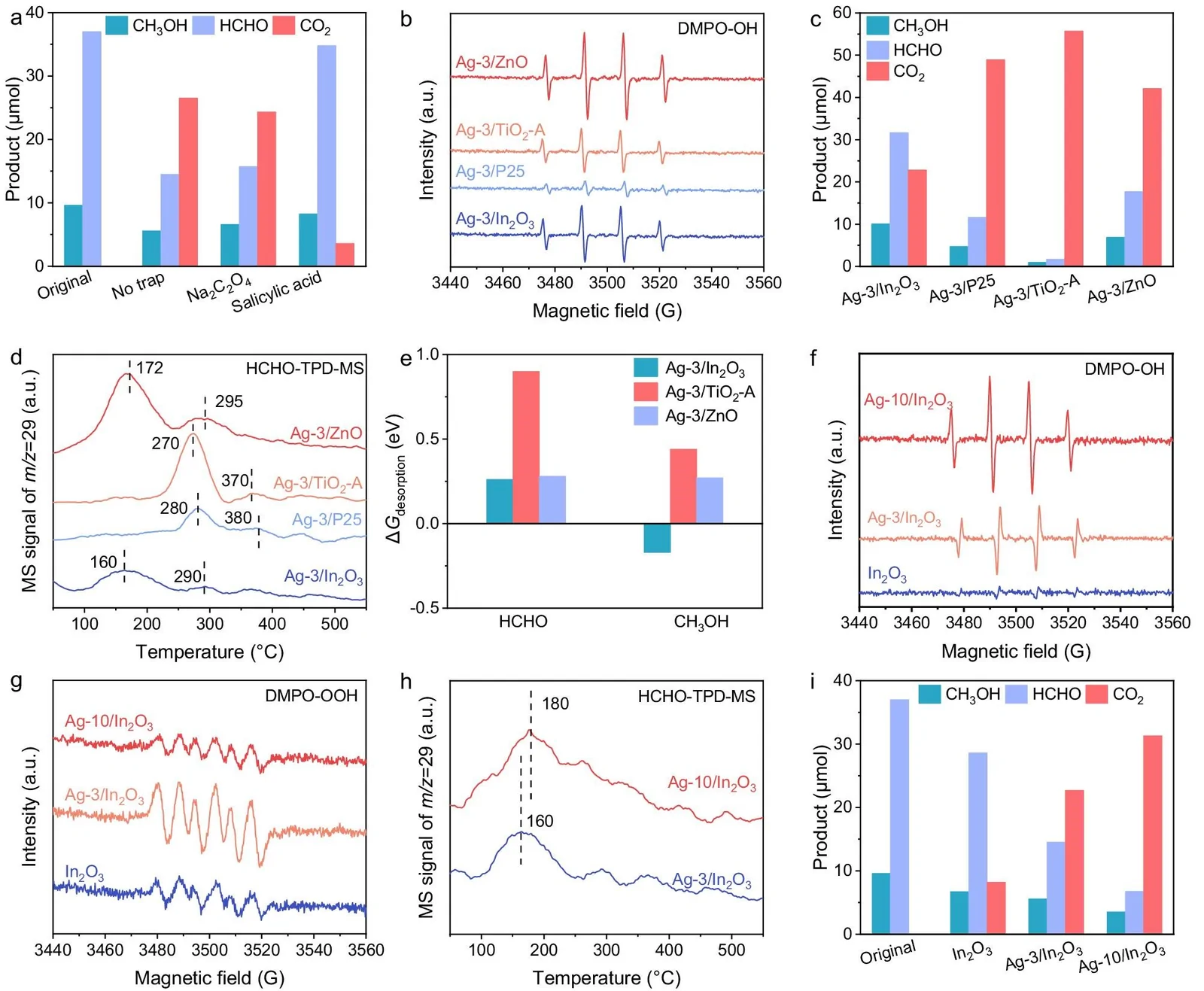
Contrast experiments combined with DFT calculations for studying the catalytic mechanism of inhibiting overoxidation. (a) Radical trapping experiments of photo–thermal catalytic HCHO oxidation over Ag-3/In_2_O_3_. (b, f and g) *In situ* EPR spectra of different catalysts under light conditions. (c and i) Photo–thermal catalytic HCHO oxidation. (d and h) HCHO-TPD-MS profiles of different catalysts. (e) Desorption free energies of CH_3_OH and HCHO on different catalysts.

After determining the catalytic function of ·OH radicals in the overoxidation reaction, *in situ* experiments were carried out to further monitor their concentration. *In situ* EPR measurements were performed using DMPO as a trapping agent, with the DMPO-·OH and DMPO-·OOH adducts serving as the respective detection signatures for the ·OH and ·OOH radicals. Thus, the comparative EPR spectra were collected under the same reaction conditions with 3 wt% Ag loaded on different oxide supports (referring to Ag-3/ZnO, Ag-3/P25, and Ag-3/TiO_2_-A), revealing that the formation of ·OH radicals on all catalysts occurs within 1 minute of light irradiation in an O_2_-saturated aqueous solution, as evidenced by a characteristic quartet signal with a 1:2:2:1 intensity ratio. Based on the signal intensity, it is inferred that the ·OH radical concentration follows the order of Ag-3/ZnO > Ag-3/In_2_O_3_ > Ag-3/TiO_2_-A > Ag-3/P25 (Fig. 4b). At the same time, the corresponding overoxidation tests upon these catalysts using CH_3_OH or HCHO as feedstock were conducted to further confirm an activity trend of Ag-3/TiO_2_-A > Ag-3/P25 > Ag-3/ZnO > Ag-3/In_2_O_3_ (Fig. 4c and Fig. S21). This discrepancy manifests that overoxidation is not solely governed by the concentration of ·OH radicals but is also critically influenced by the adsorption strength of the catalyst toward intermediates.

Hence, CH_3_OH and HCHO temperature-programmed desorption experiments coupled with mass spectrometry (CH_3_OH-TPD-MS, HCHO-TPD-MS) measurements demonstrate that Ag-3/In_2_O_3_ displays lower desorption temperatures for both HCHO and CH_3_OH relative to Ag-3/P25, Ag-3/ZnO, and Ag-3/TiO_2_-A (Fig. 4d and Fig. S22), suggesting a weaker adsorption affinity of Ag-3/In_2_O_3_ toward these two intermediates. In line with this observation, density functional theory (DFT) calculations (Fig. 4e) reveal that both CH_3_OH and HCHO possess considerably low desorption free energies on the Ag-3/In_2_O_3_ surface, rendering them prone to desorption. Consequently, such weak adsorption behavior hinders the retention of these reaction intermediates on the catalyst surface for subsequent oxidation to CO_2_, which is in good agreement with the experimentally observed low CO_2_ production activity over Ag-3/In_2_O_3_. Thus, because In_2_O_3_ exhibits weaker adsorption of CH_3_OH and HCHO compared to other supports, its use as a carrier favors the selective formation of liquid oxygenates.

In Fig. 4f, the signal intensity of DMPO-·OH over Ag-10/In_2_O_3_ is significantly stronger than that over Ag-3/In_2_O_3_ and In_2_O_3_. Semiquantitative analysis of photogenerated ·OH radicals using the coumarin reaction, which produces 7-HC, further reveals that Ag-10/In_2_O_3_ exhibits the strongest PL intensity of 7-HC, indicating its highest efficiency in generating ·OH radicals (Fig. S23a). When the experiment is conducted in a CH_3_OH solution with O_2_ under identical irradiation conditions, six prominent characteristic signals corresponding to DMPO-·OOH radicals are observed. Notably, Ag-10/In_2_O_3_ exhibits a markedly weaker signal of DMPO-·OOH compared to Ag-3/In_2_O_3_ in this configuration (Fig. 4g). Nitroblue tetrazolium chloride (NBT) photodegradation experiments, employed for semiquantitative analysis of ·OOH concentration in the system, further confirm that the ·OOH radical concentration over Ag-10/In_2_O_3_ is lower than that over Ag-3/In_2_O_3_ (Fig. S23b). These findings suggest a distinct radical generation pathway influenced by Ag loading. Given that CH_3_OOH forms mainly through the coupling of ·OOH and ·CH_3_, the lower ·OOH radical concentration of Ag-10/In_2_O_3_ leads to a sharp decline in CH_3_OOH yield. Since CH_3_OOH decomposes thermally to produce HCHO, the reduced level of this peroxide intermediate directly explains the lower HCHO output.

To elucidate why Ag-3/In_2_O_3_ exhibits high selectivity toward liquid oxygenates while Ag-10/In_2_O_3_ favors CO_2_ formation, we first compared HCHO adsorption behaviors using HCHO-TPD-MS (Fig. 4h). Ag-10/In_2_O_3_ exhibits a higher HCHO desorption temperature than Ag-3/In_2_O_3_, suggesting stronger adsorption affinity toward HCHO. The adsorbed HCHO occupies the active sites on the catalyst surface and undergoes deep oxidation. Subsequent oxidation experiments using HCHO as the reactant were conducted (Fig. 4i). Under the conditions of 37 μmol initial HCHO feedstock and 1 h light irradiation at 150°C, 14.49 μmol HCHO remains in the Ag-3/In_2_O_3_ system, with 22.6 μmol CO_2_ generated. In contrast, Ag-10/In_2_O_3_ leaves only 6.75 μmol HCHO, whereas 31.31 μmol of CO_2_ is produced. A similar trend is observed when CH_3_OH was used as the reactant (Fig. S24). These results clearly demonstrate that lower Ag loading effectively suppresses the deep oxidation of intermediate oxygenates, thereby minimizing CO_2_ formation and enhancing the selectivity toward liquid products. Combining the results presented in Fig. 4a, f, and h, it is evident that catalysts with higher Ag loading not only adsorb more HCHO but also generate a higher concentration of ·OH radicals. These two factors synergistically promote the deep oxidation pathway. As a result, Ag-3/In_2_O_3_ achieves 100% selectivity toward liquid oxygenates while maintaining a high HCHO yield, whereas the selectivity drops to 82% over Ag-10/In_2_O_3_ due to enhanced overoxidation.

To elucidate the reaction mechanism on the Ag-3/In_2_O_3_ surface, DFT calculations were performed, and the results are presented in Fig. 5. O_2_ first adsorbs onto the catalyst surface in a side-on configuration. Subsequently, CH_4_ adsorbs onto the activated *O_2_ species and is converted into *CH_3_O and *OH intermediates, which then further react to form methanol. This step is identified as the potential-determining step of the entire reaction pathway, with a Gibbs free energy change of 1.01 eV. The subsequent desorption of CH_3_OH is exothermic. The adsorbed CH_3_OH can follow two competing routes: one requires an endothermic uptake of 0.33 eV to form *CH_3_OOH, while the other exothermically produces *HCHO. The desorption of CH_3_OOH is exothermic, whereas the desorption of HCHO requires an endothermic energy of 0.13 eV. Furthermore, *CH_3_OOH can undergo further exothermic conversion to HCHO. Based on the above discussion, it can be concluded that the catalyst favors the formation of CH_3_OH and HCHO, while the yield of CH_3_OOH is expected to be significantly lower than that of the former two. This conclusion is consistent with the experimental results.

**Figure 5.**
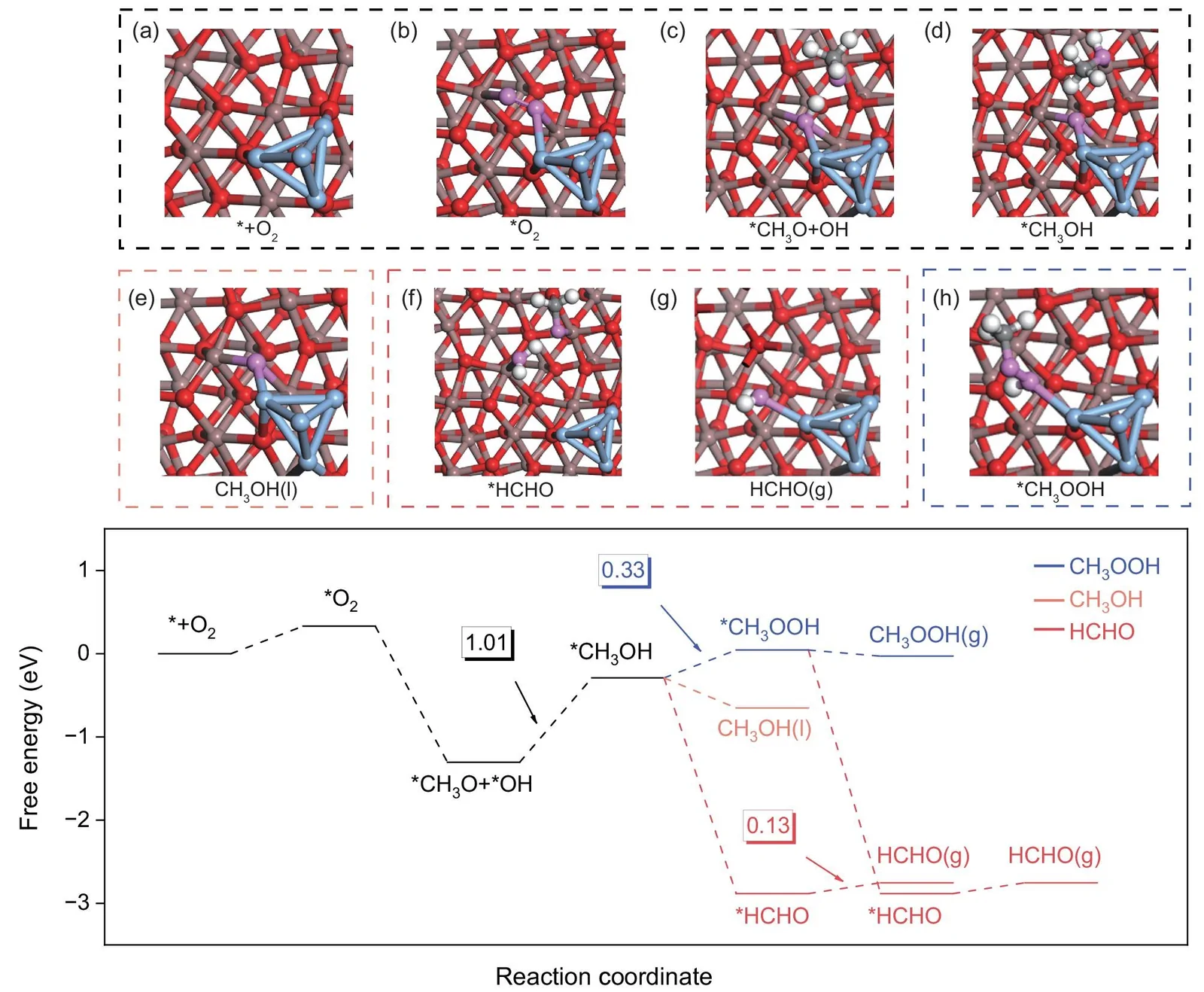
Gibbs free energy profile of the reaction pathway on Ag-3/In_2_O_3_ and the corresponding intermediate structures. Brown, red, white, light blue, and gray represent In, O, H, Ag, and C, respectively. The pink represents O in the intermediate to distinguish it from O in the catalyst.

Based on the above research, the proposed mechanism is outlined in Fig. 6 for the photo–thermal catalytic CH_4_ oxidation over Ag-3/In_2_O_3_ and Ag-10/In_2_O_3_. Under Xe lamp irradiation, photoexcitation of the In_2_O_3_ lattice generates electron–hole pairs. The e^−^ transfers to Ag nanoparticles, where they drive the reduction of O_2_ to form the ·OH and ·OOH radicals. Concurrently, h^+^ accumulates at surface sites of In_2_O_3_, activating CH_4_ via C–H bond cleavage to form the ·CH_3_ radicals. Subsequently, these radical species then undergo selective coupling: the ·CH_3_ radicals combine with the ·OH radicals to form CH_3_OH or react with the ·OOH radicals to generate CH_3_OOH. Under thermal activation at 150°C, CH_3_OOH decomposes into HCHO and CH_3_OH. Benefiting from its decreased HCHO adsorption amounts and limited generation of ·OH radicals, Ag-3/In_2_O_3_ suppresses the overoxidation of HCHO to CO_2_, which ensures high selectivity toward liquid products. On the contrary, Ag-10/In_2_O_3_ exhibits overoxidation with decreased liquid product selectivity and increased CO_2_ yields. The overall reaction pathway from CH_4_ to liquid oxygenates over Ag-3/In_2_O_3_ and Ag-10/In_2_O_3_ can be summarized by the following key equations (Equations 1–9):


(1) \begin{eqnarray*}
{\mathrm{I}}{{\mathrm{n}}}_2{{\mathrm{O}}}_3 + {{hv}}\rightarrow{\mathrm{I}}{{\mathrm{n}}}_2{{\mathrm{O}}}_3 + {{\mathrm{e}}}^ - + {{\mathrm{h}}}^ + ,
\end{eqnarray*}

(2) \begin{eqnarray*}{\mathrm{Ag}} + {{\mathrm{O}}}_2 + 2{{\mathrm{e}}}^ - + 2{{\mathrm{H}}}^ + \rightarrow{\mathrm{Ag}} + 2\cdot{\mathrm{OH}},\end{eqnarray*}

(3) \begin{eqnarray*}{\mathrm{Ag}} + {{\mathrm{O}}}_2 + {{\mathrm{e}}}^ - + {{\mathrm{H}}}^ + \rightarrow{\mathrm{Ag}} + \cdot{\mathrm{OOH}},\end{eqnarray*}

(4) \begin{eqnarray*}{\mathrm{C}}{{\mathrm{H}}}_4 + {{\mathrm{h}}}^ + \rightarrow\cdot{\mathrm{C}}{{\mathrm{H}}}_3 + {{\mathrm{H}}}^ + ,\end{eqnarray*}

(5) \begin{eqnarray*}\cdot{\mathrm{OH}} + \cdot{\mathrm{C}}{{\mathrm{H}}}_3\rightarrow{\mathrm{C}}{{\mathrm{H}}}_3{\mathrm{OH}},\end{eqnarray*}

(6) \begin{eqnarray*}\cdot{\mathrm{OOH}} + \cdot{\mathrm{C}}{{\mathrm{H}}}_3\rightarrow{\mathrm{C}}{{\mathrm{H}}}_3{\mathrm{OOH}},\end{eqnarray*}

(7) \begin{eqnarray*}{\mathrm{C}}{{\mathrm{H}}}_3{\mathrm{OOH}}\rightarrow{\mathrm{HCHO}} + {{\mathrm{H}}}_2{\mathrm{O}},\end{eqnarray*}

(8) \begin{eqnarray*}{\mathrm{C}}{{\mathrm{H}}}_3{\mathrm{OH}} + 2\cdot{\mathrm{OH}}\rightarrow{\mathrm{HCHO}} + 2{{\mathrm{H}}}_2{\mathrm{O}},\end{eqnarray*}

(9) \begin{eqnarray*}{\mathrm{HCHO}} + 4\cdot{\mathrm{OH}}\rightarrow{\mathrm{C}}{{\mathrm{O}}}_2 + 3{{\mathrm{H}}}_2{\mathrm{O}}.\end{eqnarray*}

**Figure 6.**
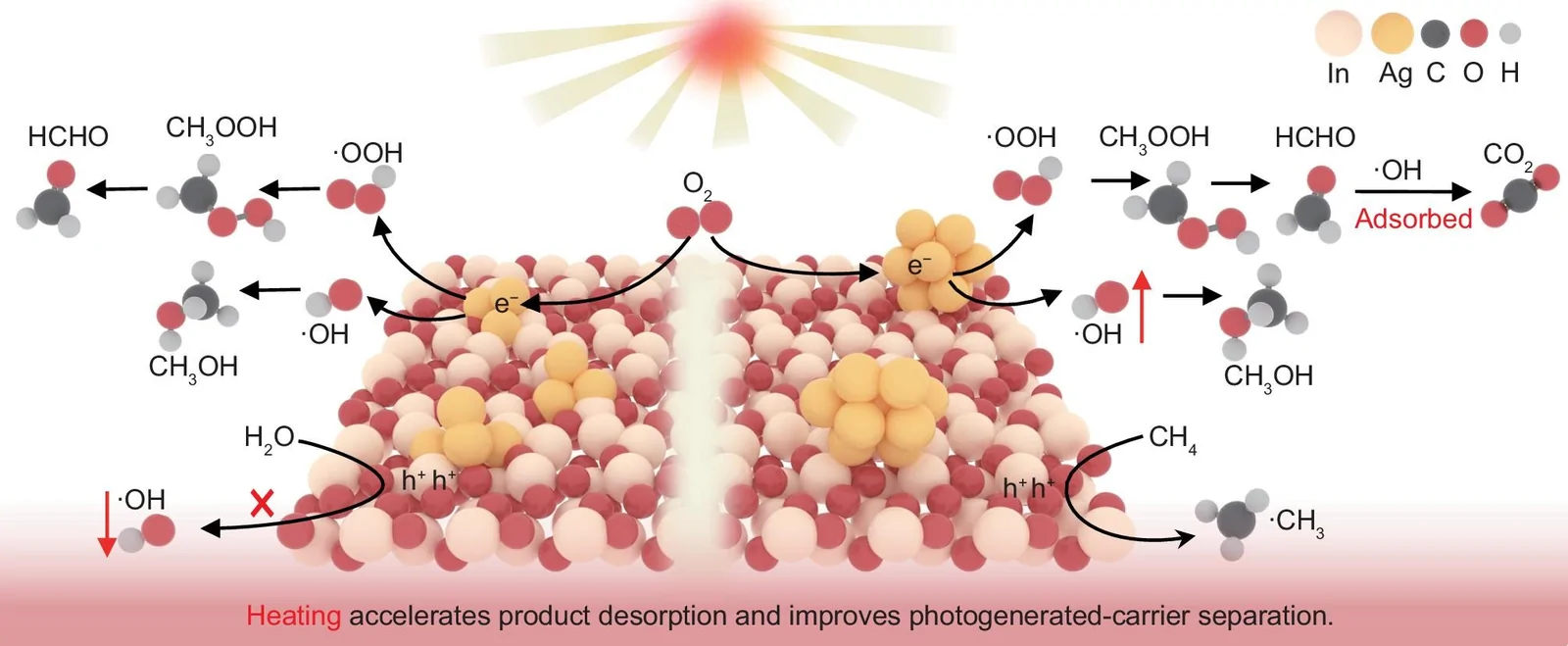
Mechanism of photo–thermal synergistic catalytic CH_4_ oxidation on Ag-3/In_2_O_3_ and Ag-10/In_2_O_3_.

## CONCLUSION

In summary, the Ag-modified In_2_O_3_ (Ag-3/In_2_O_3_) catalyst exhibits remarkable performance in the selective conversion of CH_4_ to liquid oxygenates under mild photo–thermal conditions, reaching a production rate of 6544 μmol · g^−1^ · h^−1^ with 100% selectivity. By virtue of the suitable valence band position of In_2_O_3_ support, which lies above the oxidation potential of H_2_O to ·OH radicals but below that of CH_4_ to ·CH_3_ radicals, the photogenerated holes selectively convert CH_4_ into ·CH_3_ radicals while enabling regulation of the ·OH radical concentration. Furthermore, In_2_O_3_ possesses the additional advantage of weak adsorption toward CH_3_OH and HCHO. Compared to the Ag-10/In_2_O_3_ catalyst, the low-loading Ag-3/In_2_O_3_ exhibits both decreased HCHO adsorption amounts and ·OH radical concentration, effectively suppressing overoxidation of liquid oxygenates to CO_2_. Additionally, thermal energy further contributes by accelerating reaction kinetics and promoting product desorption. By combining the advantages of photocatalysis and thermocatalysis systems, this work demonstrates a synergistic route for selective CH_4_ oxidation under mild conditions, offering a viable design strategy for advanced photo–thermal catalysts in sustainable CH_4_ valorization.

## Supplementary Material

nwag472_Supplemental_File
