# Supplementary material for: Steering the reaction pathway of methane-to-oxygenates with zero CO2 production via photo–thermal catalysis
Source: Natl Sci Rev. 2026 Aug 5;13(19):nwag472. doi: 10.1093/nsr/nwag472 (PMC13717460; doi:10.1093/nsr/nwag472)
Supplement: Supplementary file 1 [file nwag472_supplemental_file.pdf]

Supplementary Information for

Steering the reaction pathway of methane–to–oxygenates with zero CO_2 production via photo–thermal catalysis

Xia Li†, Junzhi Wang†, Fengxia Wang†, Yajie Fu, Peng Wang*, Wei Lin*, Yu Tang, Lizhi Wu and Li Tan*

State Key Laboratory of Green and Efficient Development of Phosphorus Resources, College of Chemistry, School of Future Technology, Fuzhou University, Fuzhou 350108, China

***Corresponding authors.** E-mails: p_wang@fzu.edu.cn; wlin@fzu.edu.cn; tan@fzu.edu.cn

†Equally contributed to this work.

Experimental Section

Catalyst Preparation

Materials and chemicals

Silver nitrate ($AgNO_3$, ≥99.0%, Sigma-Aldrich) was used as a metal precursor. Indium oxide (In_2O_3, 99.99% metals basis, <100 nm, Aladdin), anatase titanium dioxide (TiO_2, 99.8% metals basis, 60 nm, Aladdin), P25 titanium dioxide (20 nm, Macklin), and zinc oxide (ZnO, 99.8% metals basis, 50±10 nm, Aladdin) were used as carriers. Indium nitrate hydrate ($In(NO_3)_3·xH_2O$, 99.9% metals basis, Aladdin) and urea (H_2NCONH_2, >99%, Aladdin) were used to synthesize In_2O_3 nanorods. Sodium borohydride ($NaBH_4$, ≥98%, Aladdin) was used as a reducing agent. All chemicals were used as received without further purification. The water used in all experiments was de-ionized (DI).

Synthesis of In_2O_3 nanorods [1]

In a typical process, a mixture of 24 g urea and 4.0 g ($In(NO_3)_3·xH_2O$ was dissolved in 270 g DI water under stirring in a water bath maintained at 80°C for 14 h. After cooling to room temperature, the resulting product was collected by centrifugation and repeatedly washed with DI water until the supernatant reached PH=7. The solid was then dried under vacuum at 60°C overnight. Finally, the obtained white powder was annealed in air at 700°C for 5 h with a heating rate of $5°C·min^{-1}$. The resulting indium oxide nanorods are denoted as In_2O_3-NR.

Synthesis of Ag-*x*/In_2O_3 [2]

Ag-*x*/In_2O_3 catalysts were synthesized via a $NaBH_4$ reduction approach, where x denotes the nominal mass fraction of loaded Ag. Taking the preparation of 3 wt.% Ag-loading In_2O_3 (Ag-3/In_2O_3) as an example: 1 g of In_2O_3 nanoparticles was first dispersed in 250 mL DI water under ultrasonication. Subsequently, 6 mL of a 50 mM $AgNO_3$ aqueous solution was introduced into the suspension under continuous stirring, and the mixture was maintained under agitation for 4 h. Thereafter, 18 mL of a freshly prepared 0.2 M $NaBH_4$ solution was added dropwise, followed by continued stirring for another 2 h. The resulting solid was collected by centrifugation, thoroughly washed

with DI water, and dried under vacuum at 60°C overnight to yield a brown powder, designated as Ag-3/In_2O_3.

Synthesis of Ag-3/In_2O_3-NR, Ag-3/P25, Ag-3/TiO_2-A and Ag-3/ZnO

Ag-3/In_2O_3-NR, Ag-3/P25, Ag-3/TiO_2-A, and Ag-3/ZnO were prepared following the same synthesis procedure for Ag-3/In_2O_3, differing only in the substitution of the supporting material.

Catalyst Characterization

The crystalline structure of the catalyst was analyzed using X-ray diffraction (XRD, Rigaku Smart Lab SE, Akishima, Tokyo, Japan) equipped with Cu Kα radiation (λ=0.154 nm) and a HyPix-400 detector. The morphology and elemental distribution of the samples were analyzed using aberration-corrected transmission electron microscopy (AC-TEM) and energy–dispersive X-ray spectroscopy (EDS) mapping on a JEM-ARM300F2 microscope. Surface electronic states and chemical compositions were investigated using X-ray photoelectron spectroscopy (XPS, ESCALAB 250Xi, Thermo Scientific, USA) with an Al Kα X-ray source. Charge correction was performed by referencing the C 1*s* peak at 284.8 eV. The content of metal elements was determined by the inductively coupled plasma atomic emission spectrometry (iCAP 7000 Plus Series ICP–OES).

Optical properties were examined using UV-vis diffuse reflectance spectroscopy (DRS) on a Shimadzu UV-3600 spectrophotometer, with $BaSO_4$ as the reference material, covering a wavelength range of 200–800 nm. Photoluminescence (PL) spectra were acquired at room temperature using an FL/FS-900 spectrometer. Electrochemical properties, including electrochemical impedance spectroscopy (EIS), photocurrent response, and Mott–Schottky analysis, were measured using a Bio-Logic electrochemical workstation with a 0.2 M Na_2SO_4 electrolyte. A three-electrode configuration was employed, comprising a saturated Ag/AgCl reference electrode, a platinum counter electrode, and an FTO-coated conductive glass working electrode.

Electron paramagnetic resonance (EPR) spectroscopy was performed on a Bruker EMXplus spectrometer (Germany) to detect radical species. The catalyst (5 mg) was

dispersed in water or methanol and mixed with 5,5–dimethyl–1–pyrroline–N–oxide (DMPO) as the radical trap. Signals corresponding to ·OH, ·OOH, and $\cdot CH_3$ radicals were recorded immediately after 1 min of xenon lamp irradiation.

In situ diffuse reflectance infrared Fourier transform spectroscopy (*In situ* DRIFTS) was conducted using a Thermo Fisher Scientific iS50 FTIR spectrometer equipped with a mercury cadmium telluride (MCT) detector and a Harrick temperature control system. For *in situ* DRIFTS, the background spectrum was collected at 30°C under an Ar atmosphere with the Xe lamp on. CH_4 was introduced for 30 min, followed by O_2 in the presence of H_2O vapor. The temperature then increased from 30 to 150°C at 5°C/min, and IR spectra were continuously recorded at 150°C until no further changes were observed.

HCHO temperature-programmed desorption coupled with mass spectrometry (HCHO-TPD-MS) was recorded by using the AutoChem II 2920 (Micromeritics Instrument Corporation). The sample (0.2 g) was pretreated in pure He at a flow rate of 50 mL min^{-1} at 350°C for 1 h. After cooling to 30°C, adsorption was performed with HCHO-saturated water vapor for 2 h. Then, the temperature was increased from 50°C to 800°C at a heating rate of 10°C/min with the carrier gas He.

CH_3OH temperature-programmed desorption coupled with mass spectrometry (CH_3OH-TPD-MS) was recorded by using the AutoChem II 2920 (Micromeritics Instrument Corporation). The sample (0.2 g) was pretreated in pure He at a flow rate of 50 mL min^{-1} at 350°C for 1 h. After cooling to 30°C, adsorption was performed with CH_3OH-saturated water vapor for 2 h. Then, the temperature was increased from 50°C to 800°C at a heating rate of 10°C/min with the carrier gas He.

Catalytic Performance Test

The catalytic activity evaluation was conducted in a photo–thermal reactor system integrated with a xenon lamp **(Figure S4)**. In a typical procedure, 5 mg of the catalyst was uniformly dispersed in 20 mL of distilled water via ultrasonication for 3 minutes. The batch reactor was then subjected to multiple cycles of O_2 purging through inflation and deflation to eliminate any residual air. Following this, the reactor was pressurized

with 1 bar of O_2, and CH_4 was introduced to achieve the desired pressure. The reaction was carried out at 150°C under full-spectrum irradiation for 1 hour. Upon completion, the reactor was rapidly cooled to below 10°C using an ice-water bath.

The quantification of CH_3OOH and CH_3OH was performed using water-suppressed pulsed ^1H nuclear magnetic resonance (NMR, AVANCE NEO, 600 MHz), with 4,4–dimethyl–4–silapentane–1–sulfonic acid (DSS) as the internal standard. Specifically, 540 μL of the post-reaction solution was mixed with a 0.1% (w/w) DSS solution. Calibration curves **(Figure S5)** were generated by correlating the area ratio of the product peaks to DSS with the known concentrations of standard solutions.

For the determination of HCHO, the acetylacetone colorimetric method was employed using a UV-2700 spectrophotometer (Shimadzu). A mixture of 3 mL of water, 2 mL of 0.25% (v/v) acetylacetone solution, and 700 μL of the product was heated in a water bath at 80°C for 1 hour. The UV-vis absorption spectrum was subsequently measured at 412 nm, and the HCHO concentration was determined based on a pre-established calibration curve **(Figure S6)**.

Gas-phase CO_2 was analyzed using a gas chromatograph (GC-2014, Shimadzu). This comprehensive analytical approach ensured accurate quantification of both liquid and gaseous products, providing a detailed assessment of the catalytic performance **(Figure S7)**.

Trapping Experiments [3]

Active species trapping experiments were conducted to investigate the mechanisms of photo–thermal catalytic CH_4 conversion. Salicylic acid and $Na_2C_2O_4$ were introduced into the reaction system to trap ·OH and h^+ active species, respectively. In a typical experiment, 5 mg of catalyst was dispersed in 20 mL of deionized water containing 1 mM of salicylic acid and $Na_2C_2O_4$ by ultrasonication for 3 min. The batch reactor was then purged with O_2 by repeated cycles of inflation and deflation to remove residual air. Subsequently, 39 bars of N_2 and 1 bar of O_2 were injected into the reactor. The reaction was carried out under xenon lamp illumination at 150°C for 1 h.

Photo–thermal Catalytic Oxidation of CH_3OH or HCHO

In the experiment, 5 mg of catalyst was dispersed in 20 mL of a CH_3OH solution or a HCHO solution by ultrasonication for 3 min. The batch reactor was then purged with O_2 by repeated cycles of inflation and deflation to remove residual air. Subsequently, 39 bars of N_2 and 1 bar of O_2 were injected into the reactor. The reaction was carried out under xenon lamp illumination at 150°C for 1 h. The detection methods for the products were the same as described above.

Analysis of Photogenerated Hydroxyl Radicals (·OH) [3]

Coumarin was employed as a fluorescent probe to detect ·OH radicals by monitoring the formation of 7-hydroxycoumarin (7-HC). Typically, 5 mg of the catalyst was dispersed in 20 mL of an aqueous coumarin solution (0.5 mM) and stirred under light irradiation for 1 h. After the reaction, the solution was sampled, filtered, and its photoluminescence (PL) spectrum was recorded using an F4500 spectrofluorometer.

Analysis of Photogenerated Superoxide Radicals (·OOH) [3]

Nitroblue tetrazolium chloride (NBT) was utilized as the probe molecule to detect ·OOH radicals. Typically, 5 mg of the catalyst was dispersed in 20 mL of an aqueous coumarin solution (0.02 mM) and stirred under light irradiation for 1 h. After the reaction, UV-vis spectra were measured using a spectrophotometer (UV-2700, Shimadzu, Japan), and the variations in the peak value at 260 nm were recorded.

Computational details

The Vienna *ab initio* simulation package (VASP) [4,5] was used for all spin-polarized DFT calculations. The exchange-correlation energy contributions were described by the Perdew-Burke-Ernzerhof (PBE) [6] functional, and the electron-ion interactions were treated by the projector augmented wave (PAW) pseudopotentials [7]. To account for van der Waals interactions, the Grimme D3 functional was used [8], and the dipole correction in the surface normal direction was applied. The kinetic cut-off energy of the plane-wave expansion was set to 450 eV. The Brillouin zone integration was sampled using the Monkhorst-Pack scheme [9]. The energy change and maximum

force convergence thresholds were set to 10^{-4} eV and 0.02 eV/Å, respectively. The real solvent environment was modeled using the conductor-like screening model (COSMO) with a dielectric constant of 78.4.[10] The thickness of the vacuum region between the repeated slabs was set to 15 Å. The atoms in the top two layers were relaxed in all directions during structural optimization, whereas the remaining atoms were fixed in their bulk positions. The change of Gibbs free energy (ΔG) was computed as follows:

$$\Delta G = \Delta E + \Delta E_{\mathrm{ZPE}} - T\Delta S$$

where ΔE, ΔE_{ZPE}, and ΔS signify the difference value of total energy, zero potential energy, and entropy, respectively, and T is the temperature. ΔE_{ZPE} and ΔS can be obtained from the vibrational frequencies under the harmonic approximation and negligible contributions of the slab.[11]

Supplementary Figures and Tables

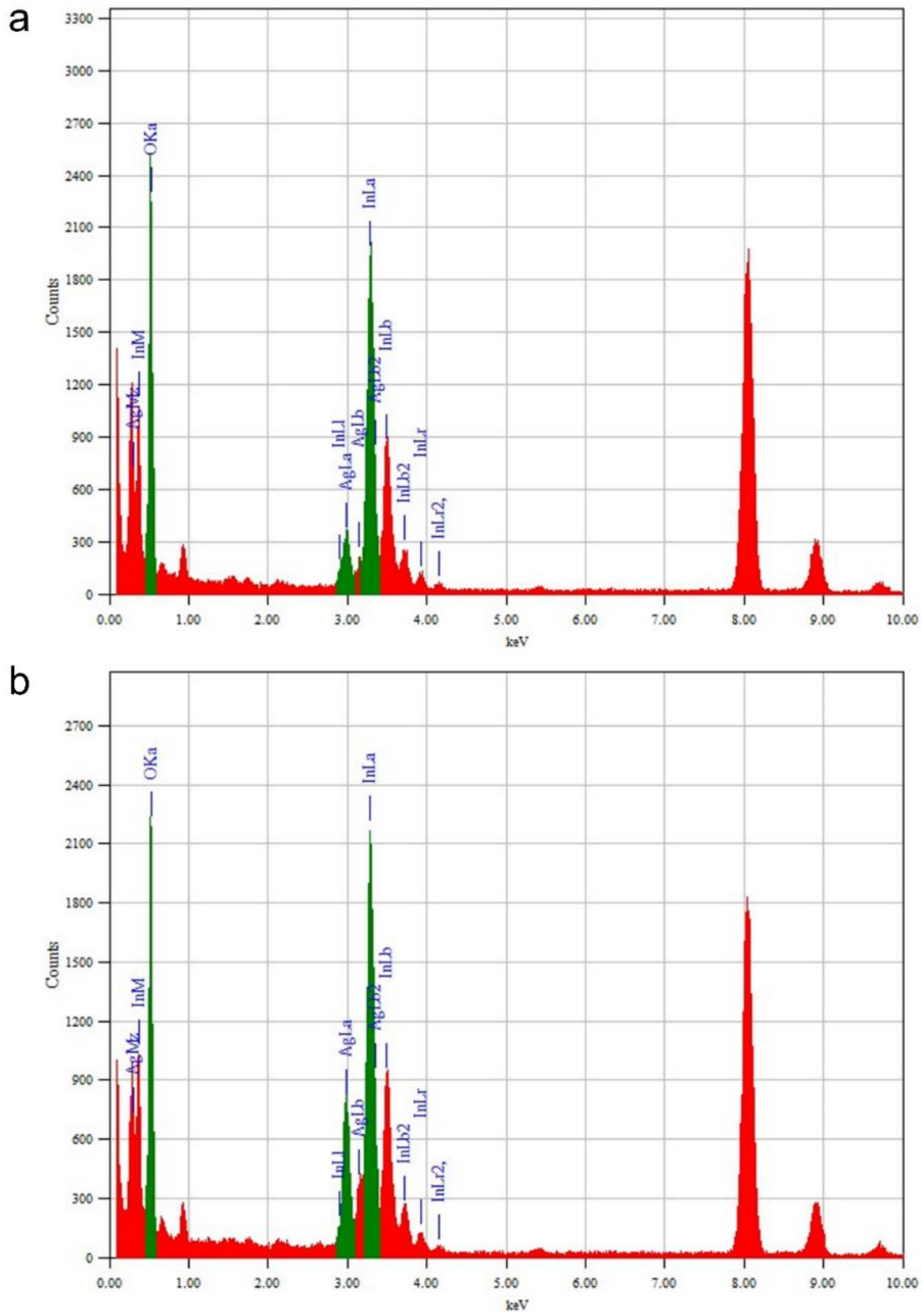

Figure S1. EDS spectra of (a) Ag-3/In_2O_3 and (b) Ag-10/In_2O_3.

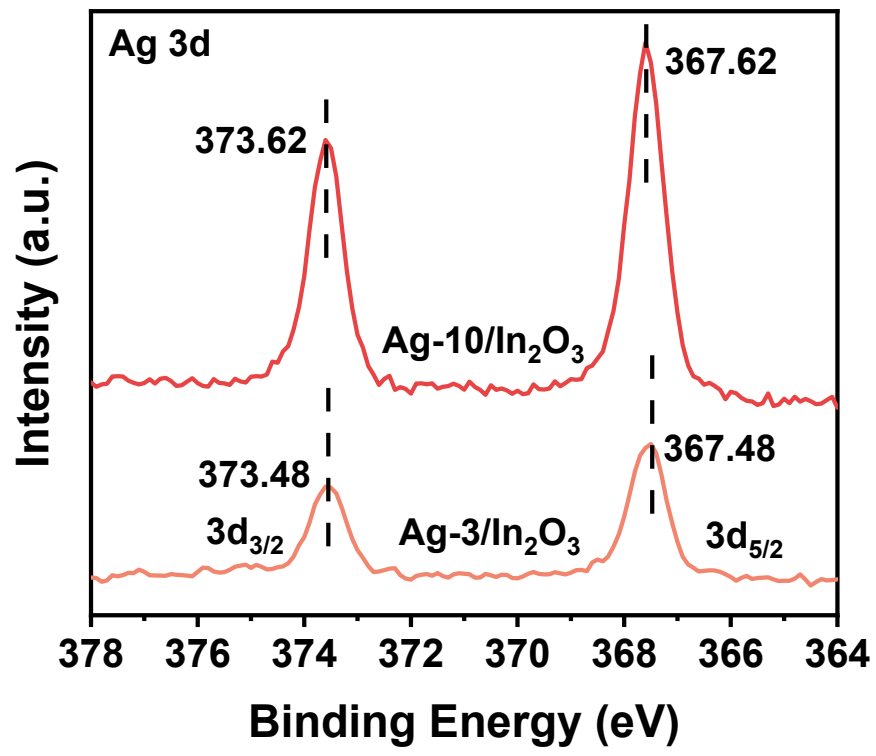

Figure S2. XPS spectra of catalysts.

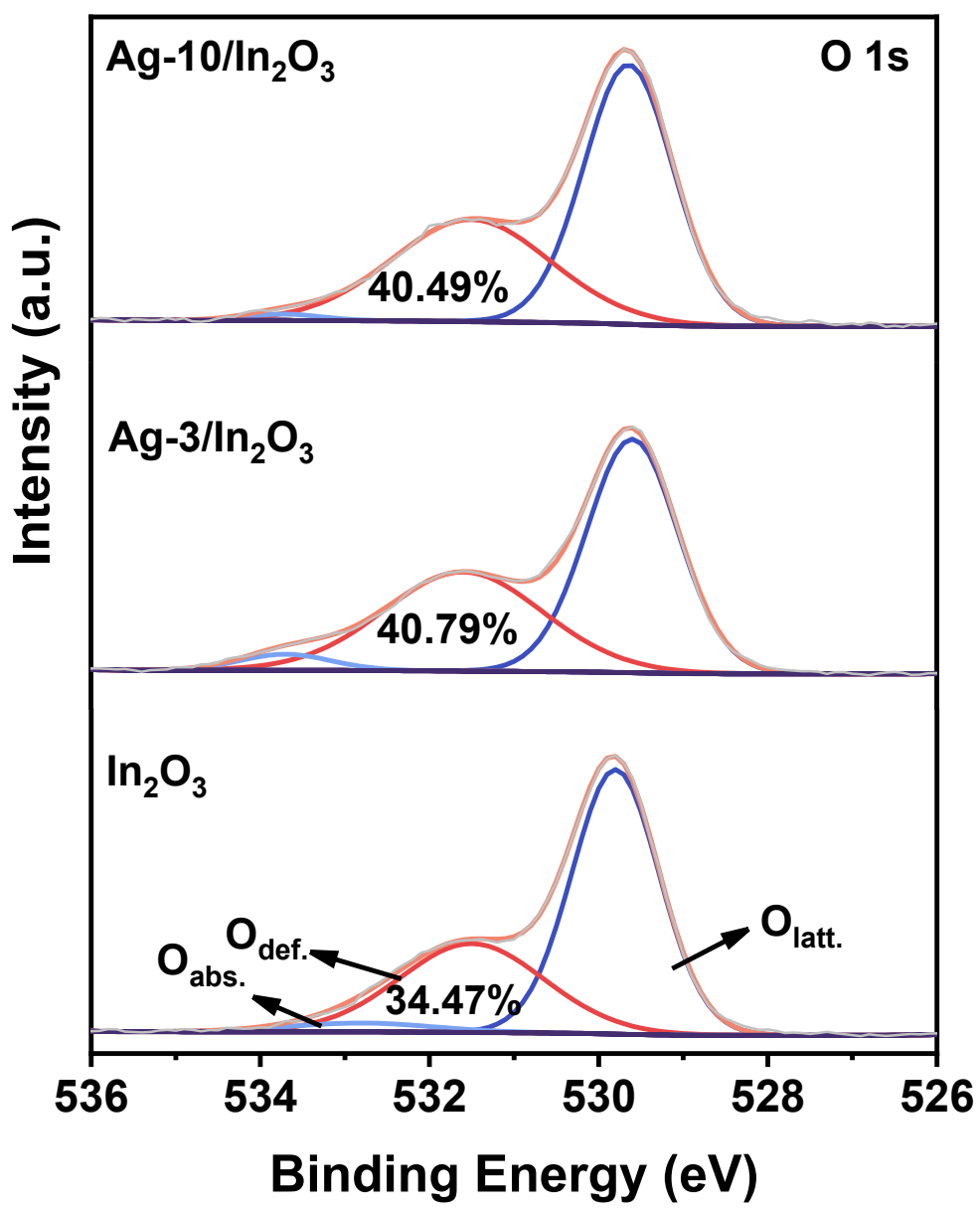

Figure S3. XPS spectra of catalysts.

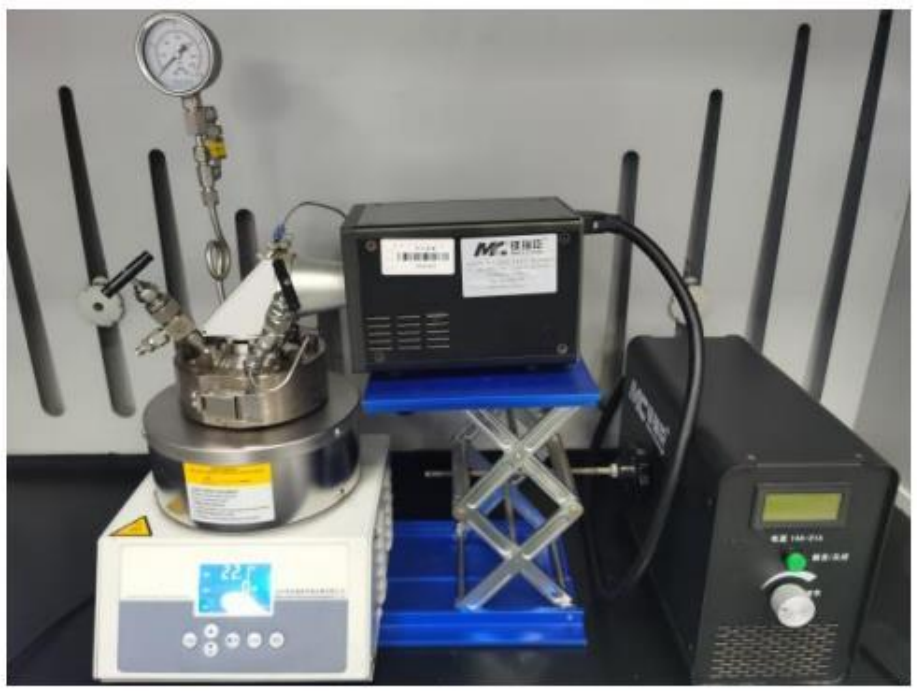

Figure S4. Illustration of photo–thermal catalytic CH_4 oxidation reaction setup.

The photo–thermal catalytic CH_4 oxidation system comprises a xenon lamp, a heating jacket, a high-pressure reactor, and a thermocouple to monitor the solution temperature in real time during the reaction. Inside the reactor, a quartz liner is used to contain the catalyst and water. The catalyst is exposed to external light through a quartz window positioned at the top, and the heating jacket provides additional thermal energy. To achieve precise temperature measurement, the temperature sleeve is completely immersed in the reaction solution, allowing the thermocouple to measure the actual temperature of the reaction mixture accurately.

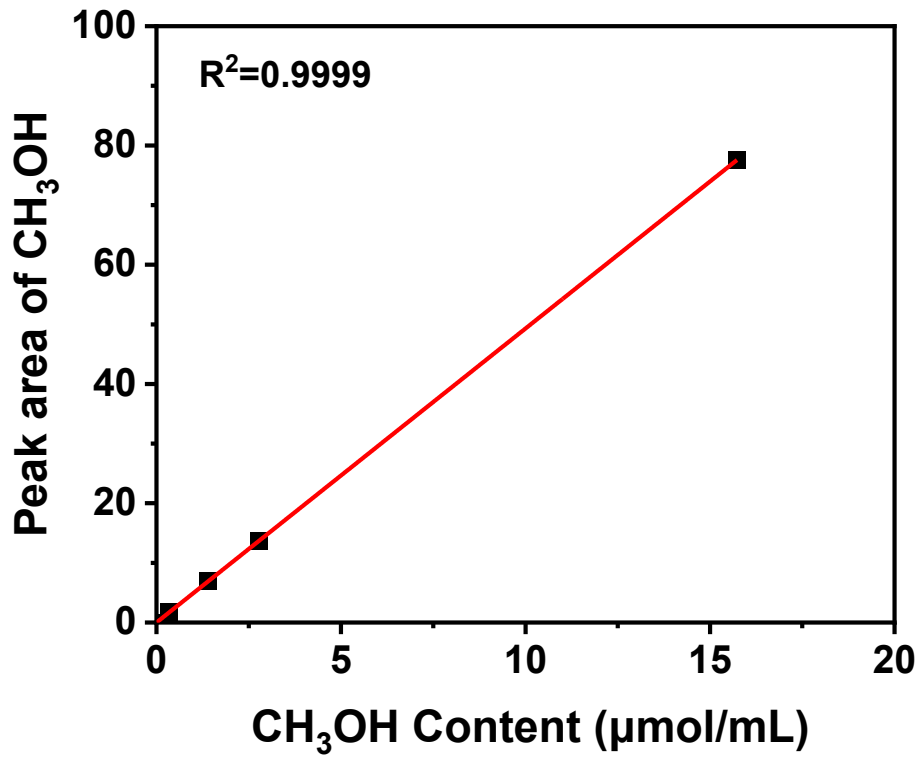

Figure S5. Calibration curve for quantification of CH_3OH by ^1H NMR. The standard curve for CH_3OOH is the same as that for CH_3OH.

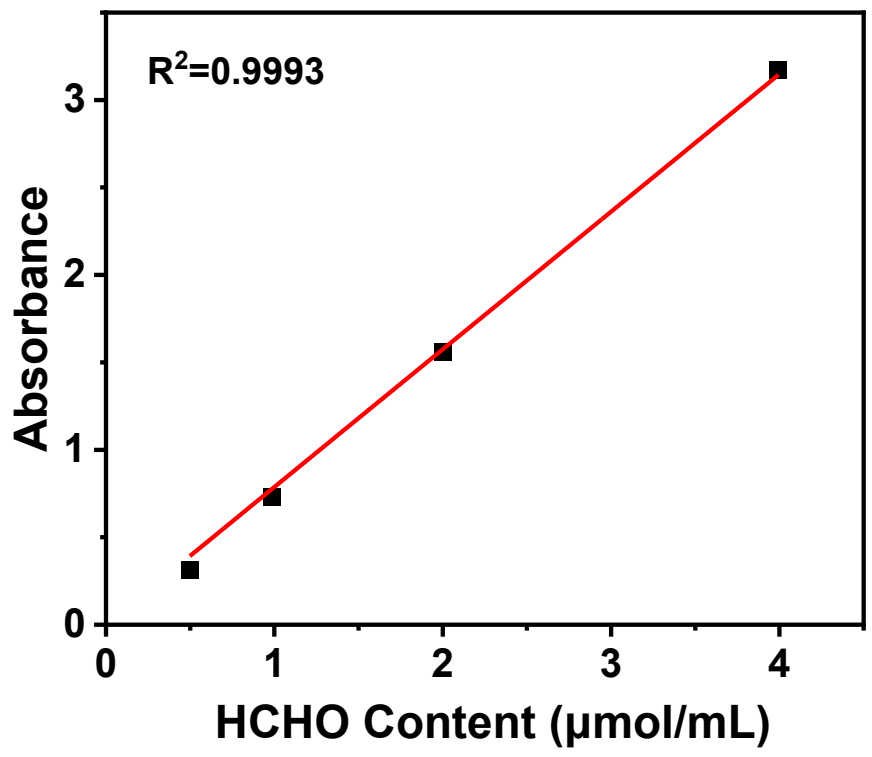

Figure S6. Calibration curve for quantification of HCHO by the colorimetric method.

The production of HCHO in water is assessed through the acetylacetone color-developing method. In the acetic acid-ammonium acetate buffer solution (pH=6), HCHO cross-links with acetylacetone and ammonia to form yellow-colored 3,5–diacetyl–1,4–dihydropyridine, exhibiting a characteristic UV-vis absorption at 412 nm. **Figure S6** manifests that the concentration of HCHO is proportional to the peak intensity at 412 nm.

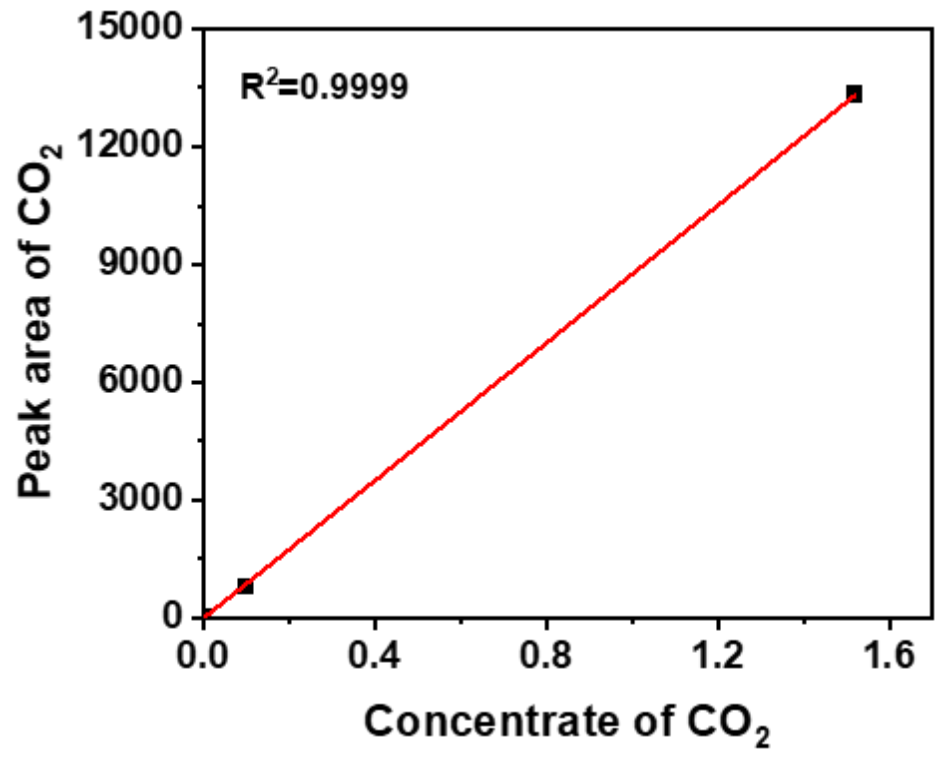

Figure S7. Standard curve for quantification of CO_2 using gas chromatography.

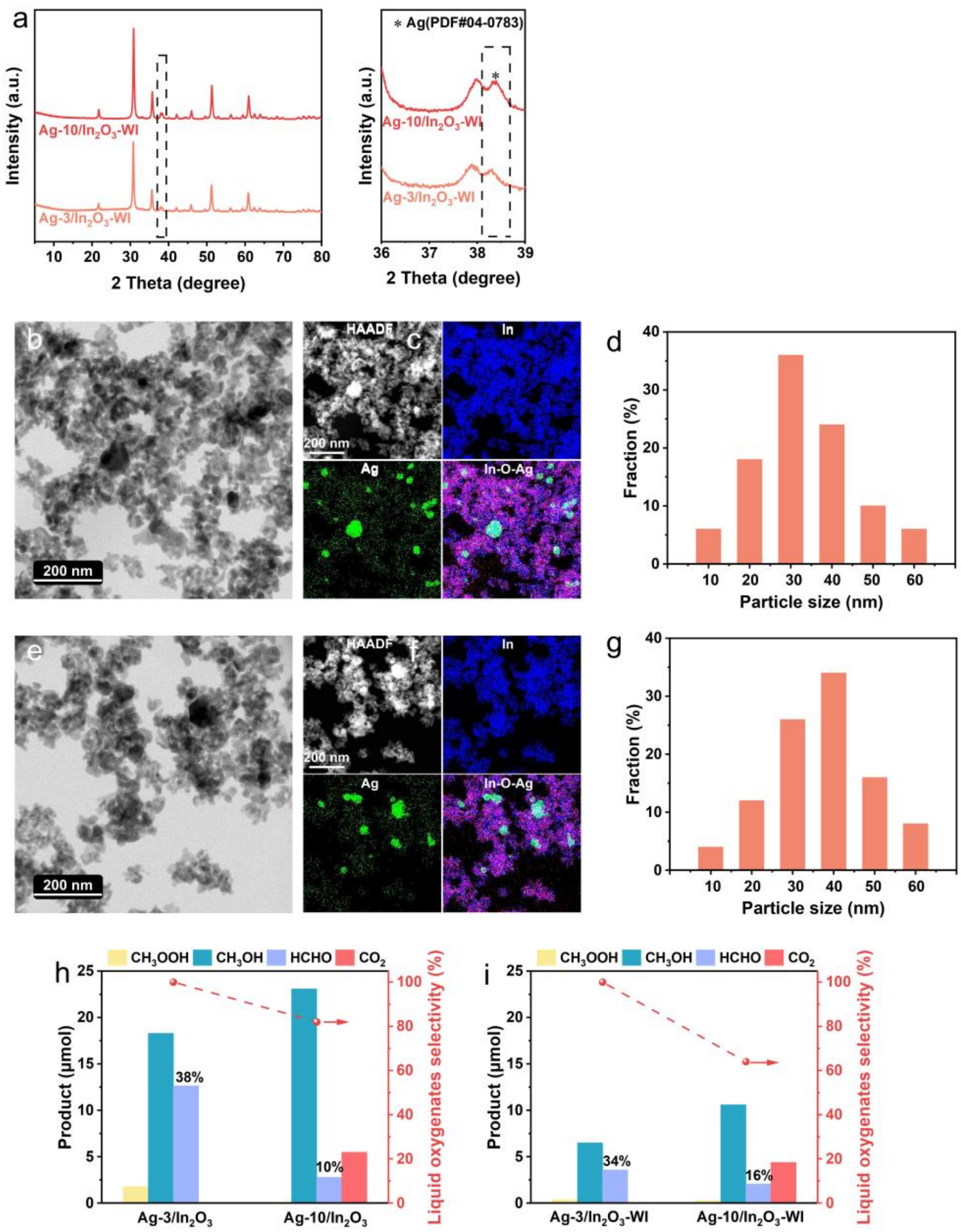

Figure S8. The XRD spectra of (a) Ag-3/In_2O_3-WI and Ag-10/In_2O_3-WI; AC-STEM images of (b, c) Ag-3/In_2O_3-WI and (e, f) Ag-10/In_2O_3-WI with corresponding EDX elemental mapping images; Statistical analysis of particle size distribution of Ag nanoparticles in (d) Ag-3/In_2O_3-WI and (g) Ag-10/In_2O_3-WI; Catalytic performance of Ag-*x*/In_2O_3 prepared by (h) $NaBH_4$ reduction method and (i) wet impregnation method.

To distinguish the effects of particle size and Ag loading amount, Ag nanoparticles with uniform size were loaded onto In_2O_3 via a wet impregnation method, successfully affording Ag-3/In_2O_3-WI and Ag-10/In_2O_3-WI catalysts for comparison. As shown in **Figure S8a**, the diffraction peak intensity of Ag at 38.1° exhibits only a tiny increase for the Ag-10/In_2O_3-WI sample compared to Ag-3/In_2O_3-WI, suggesting that the Ag

species remain well dispersed even at higher loading levels, without significant aggregation. Furthermore, the AC-STEM images present in **Figure S8b–g** reveal no obvious agglomeration of Ag nanoparticles in either the Ag-3/In_2O_3-WI or Ag-10/In_2O_3-WI samples. Statistical analysis of particle size distributions indicates that the Ag nanoparticles of both samples show a relatively consistent particle size distribution range of 10–60 nm. The slightly increased average particle size for Ag-10/In_2O_3-WI is ~40 nm, while that of Ag-3/In_2O_3-WI is ~30 nm, due to the distribution of Ag nanoparticles with higher density leading to the spatial dislocation and coincidence error in electron microscope statistics. At the same time, the almost uniform size distribution of both loading samples can be used to eliminate the particle size effect.

Collectively, the XRD patterns, AC-STEM images, and particle size statistics consistently demonstrate that the wet impregnation method produces almost uniformly sized and well-dispersed Ag nanoparticles across different Ag loadings, as well as the relatively consistent particle size distribution range, validating our approach to decouple the effects of particle size and loading in this system.

As shown in **Figures S8h and S8i**, compared with the Ag-10/In_2O_3 catalyst prepared by the $NaBH_4$ reduction method, the Ag-10/In_2O_3-WI catalyst synthesized by wet impregnation exhibits significantly lower catalytic activity, but a similar product selectivity distribution trend. Therefore, these results indicate that the Ag loading amount is the dominant factor governing product selectivity, whereas Ag particle size mainly affects catalytic activity.

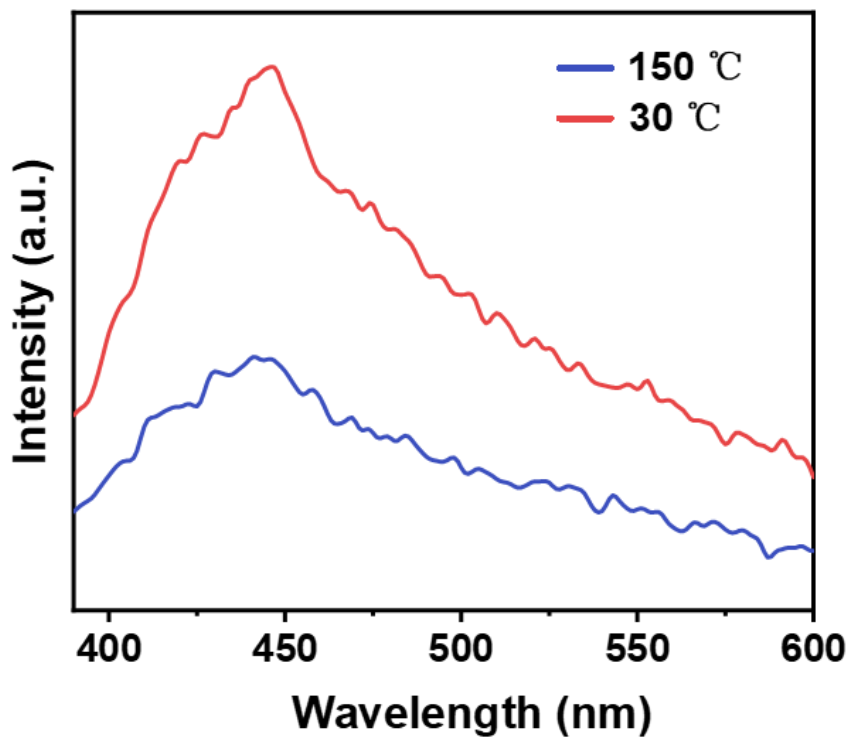

Figure S9. *In situ* PL spectra of Ag-3/In_2O_3.

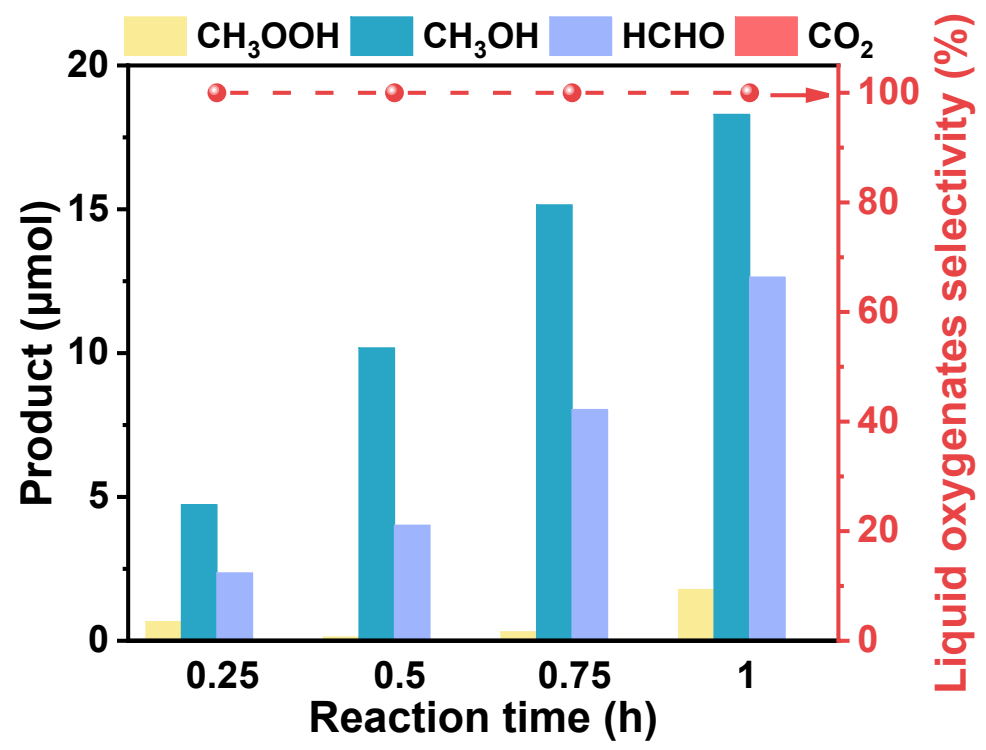

Figure S10. Photo–thermal catalytic CH_4 oxidation performance over different reaction times. Reaction conditions: Xenon lamp (320–780 nm), 5 mg catalyst, 39 bar CH_4, 1 bar O_2, 150ºC, 20 mL H_2O.

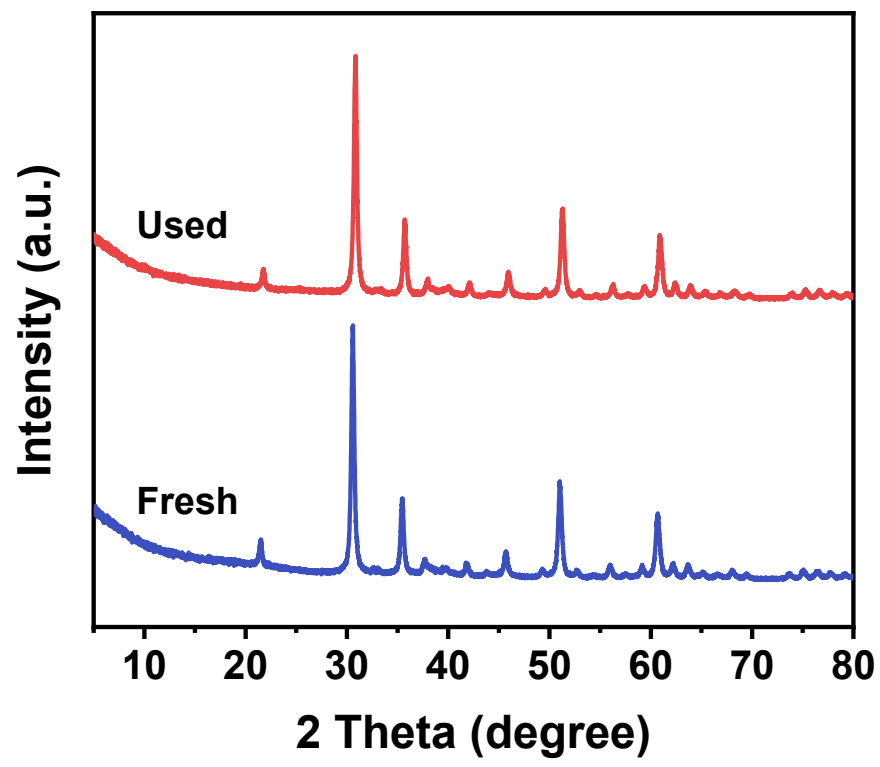

Figure S11. XRD patterns of Ag-3/In_2O_3 before and after the reaction.

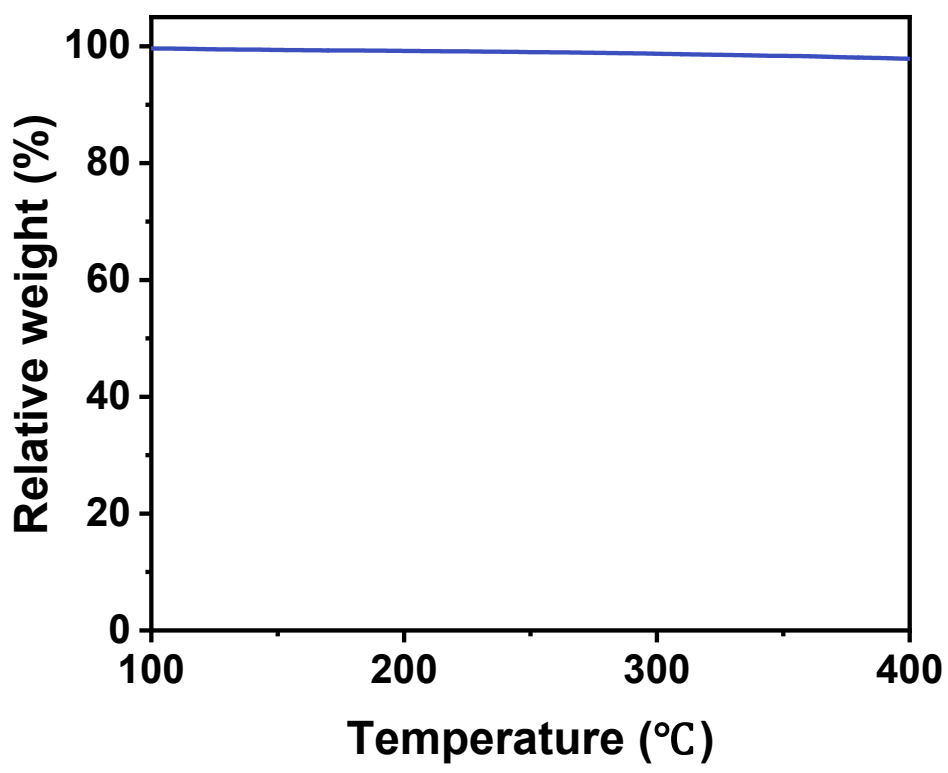

Figure S12. TG curve of Ag-3/In_2O_3 after the reaction.

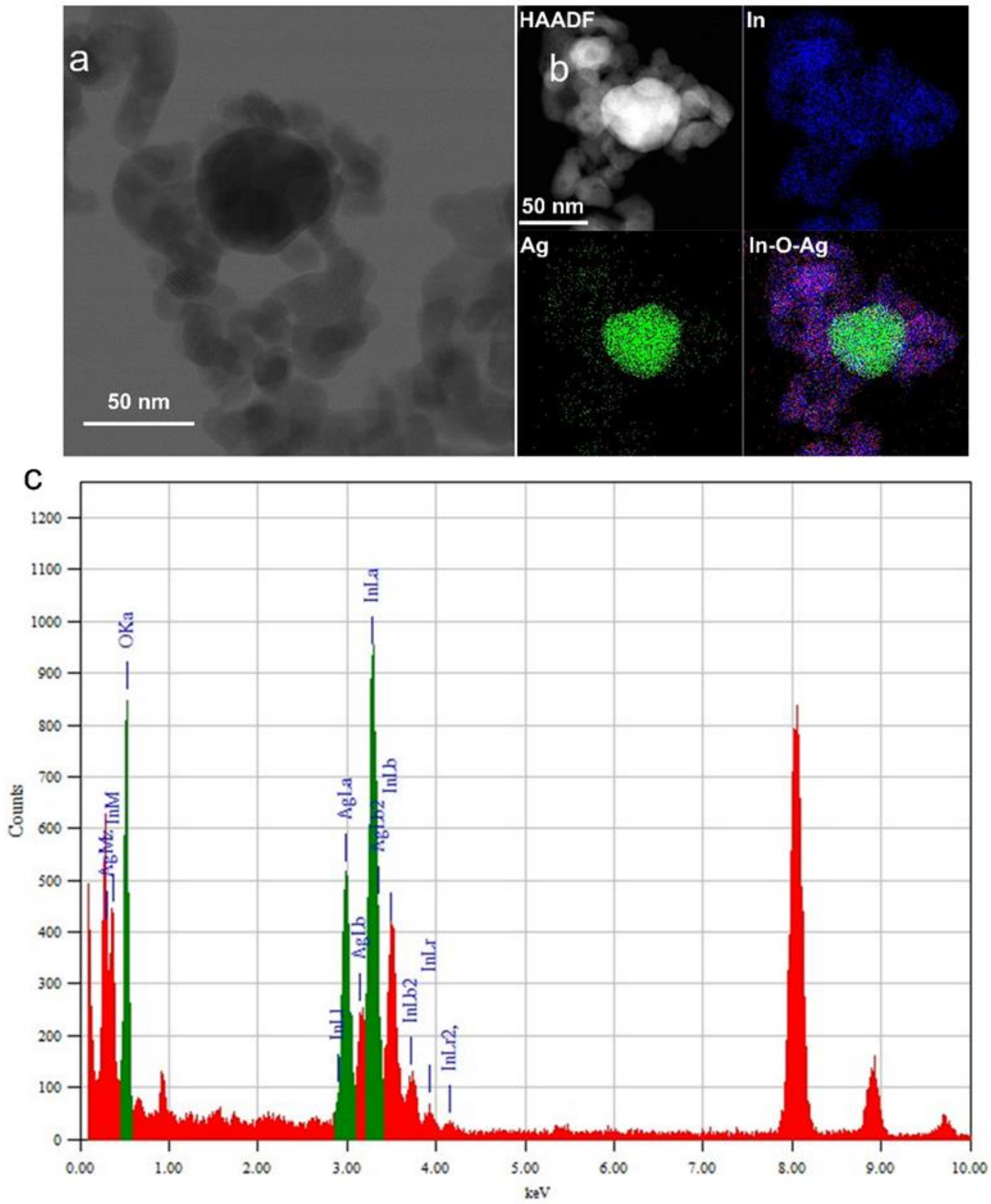

Figure S13. (a) AC-STEM image; (b) EDX elemental mapping image; (c) EDS spectrum of Ag-3/In_2O_3 after the reaction.

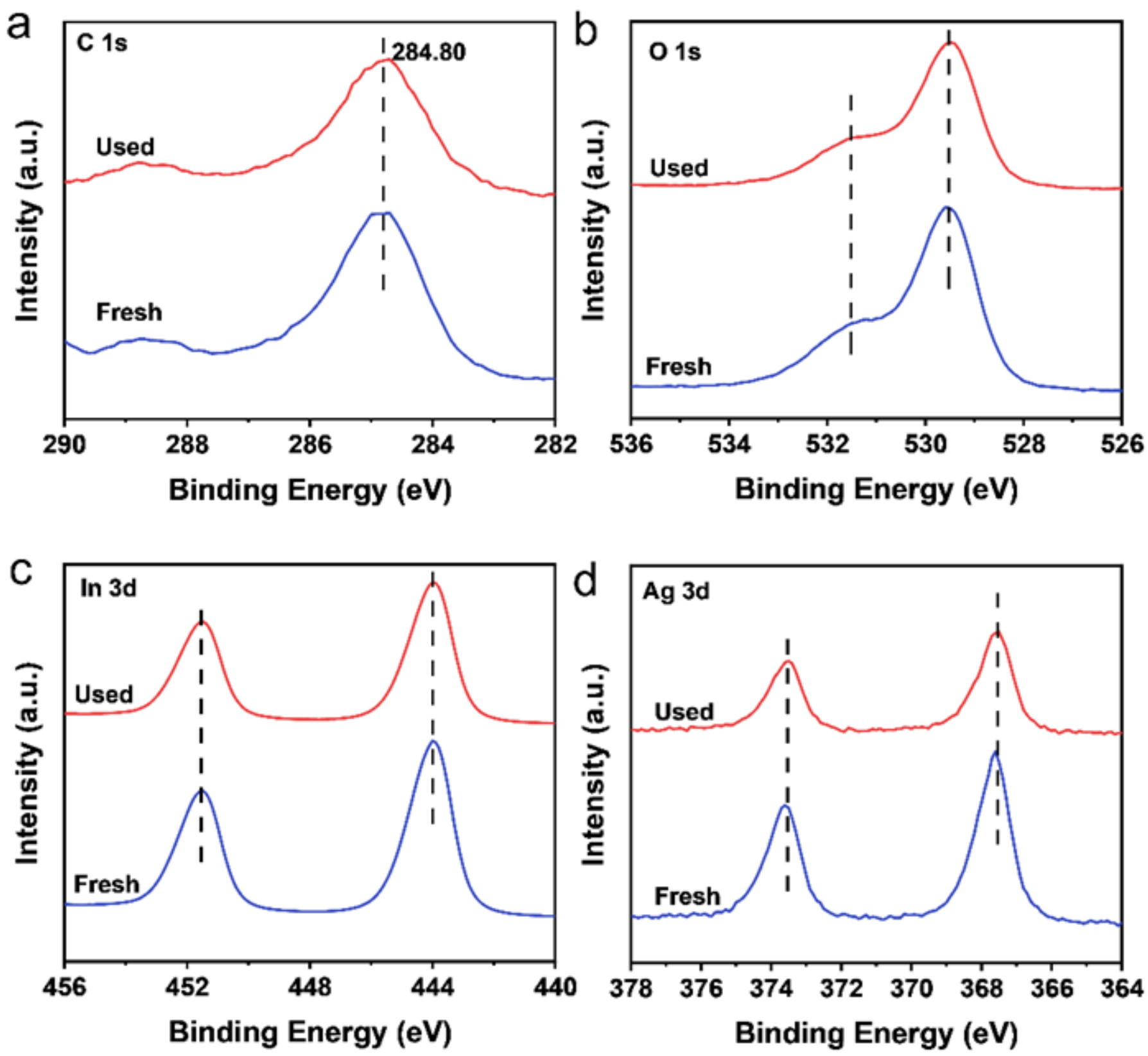

Figure S14. XPS spectra of Ag-3/In_2O_3 before and after the reaction.

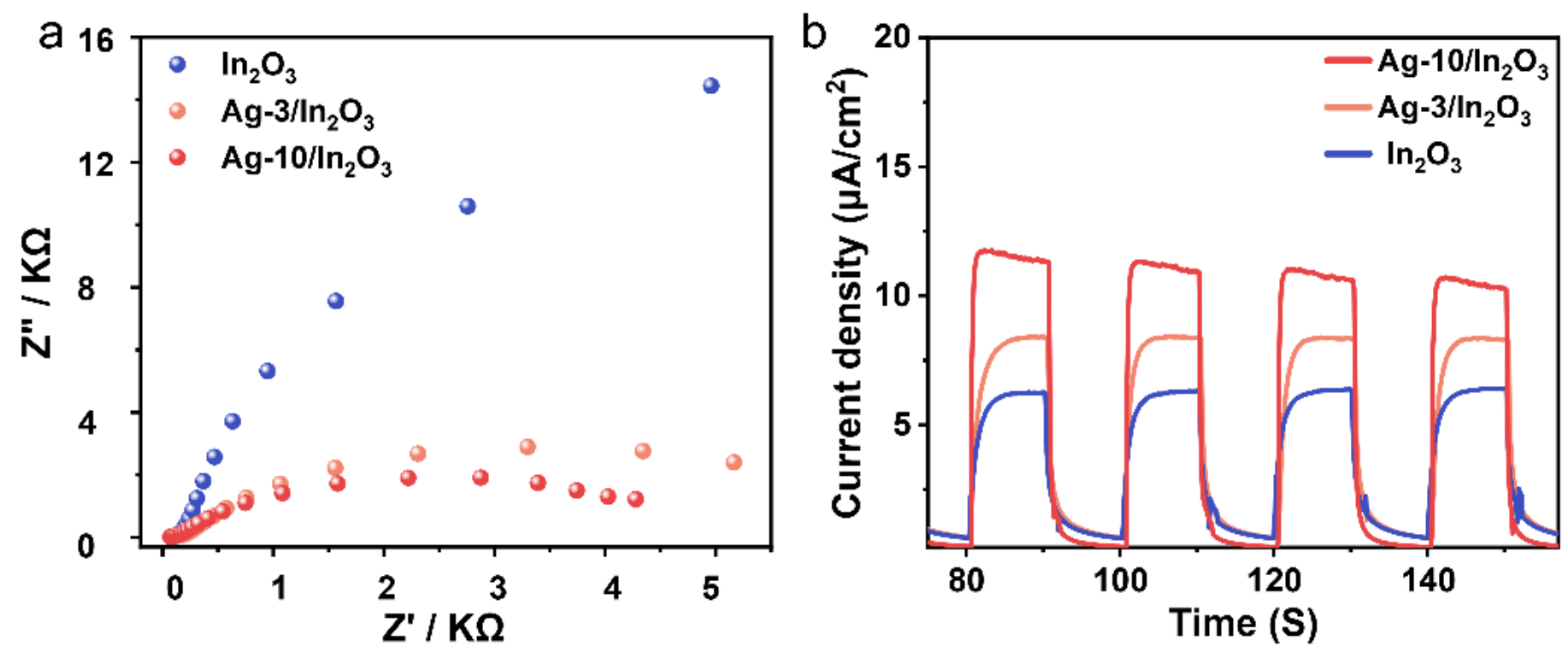

Figure S15. (a) Electrochemical impedance spectra and (b) Photocurrent spectra of catalysts.

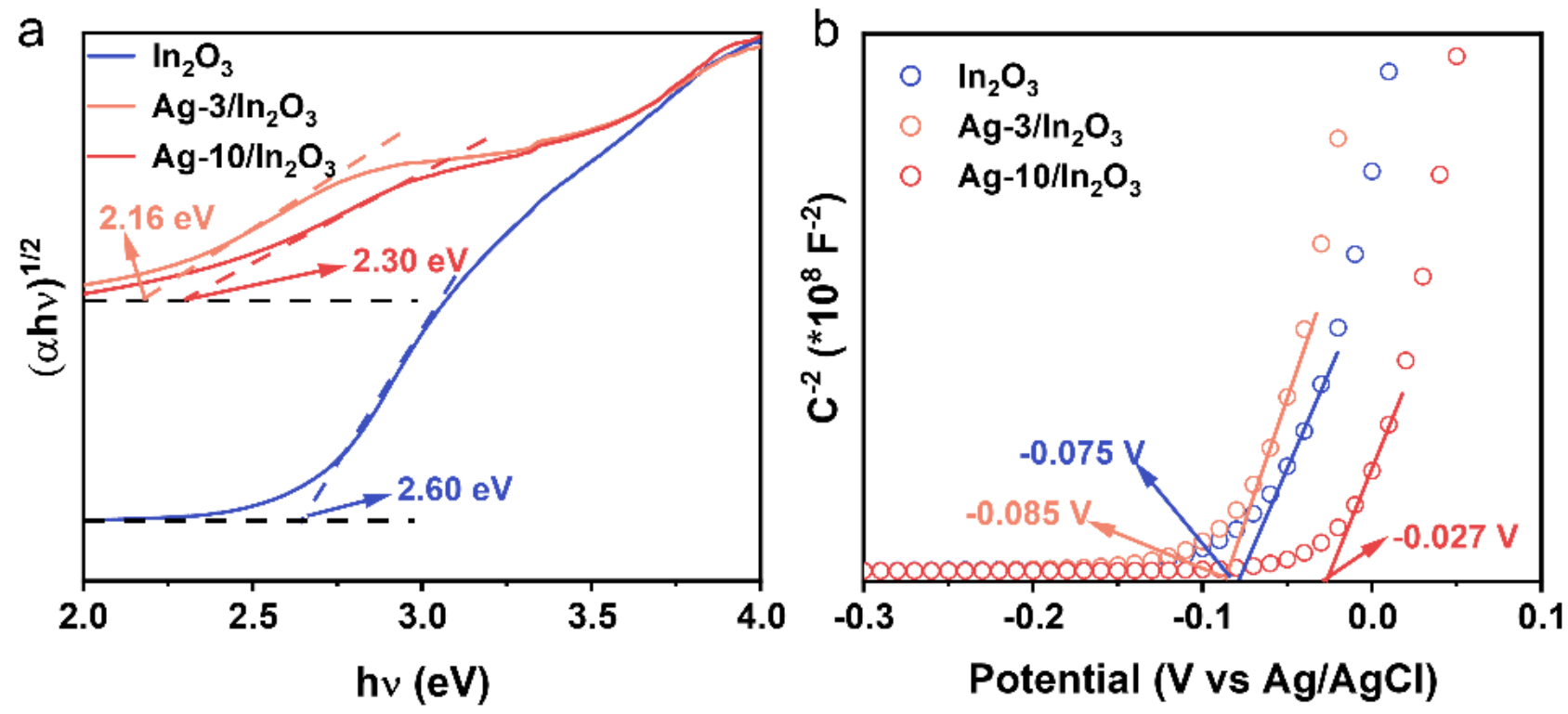

Figure S16. (a) UV-vis absorption spectra corresponding to Tauc plots of catalysts; (b) The Mott–Schottky plots of catalysts.

Based on UV-vis DRS data, we could calculate the bandgap energy (E_g) using the Kubelka–Munk transformation. And the Mott–Schottky analysis reveals the flat potential (V_{fb}). In addition, the plots with the positive slopes verify the n-type nature of In_2O_3, whose conduction band (E_{CB}) is usually more negative (0.2 V) than V_{fb}. Using the relation E_{CB}=E_{VB}+E_g, the valence band (E_{VB}) position is calculated.

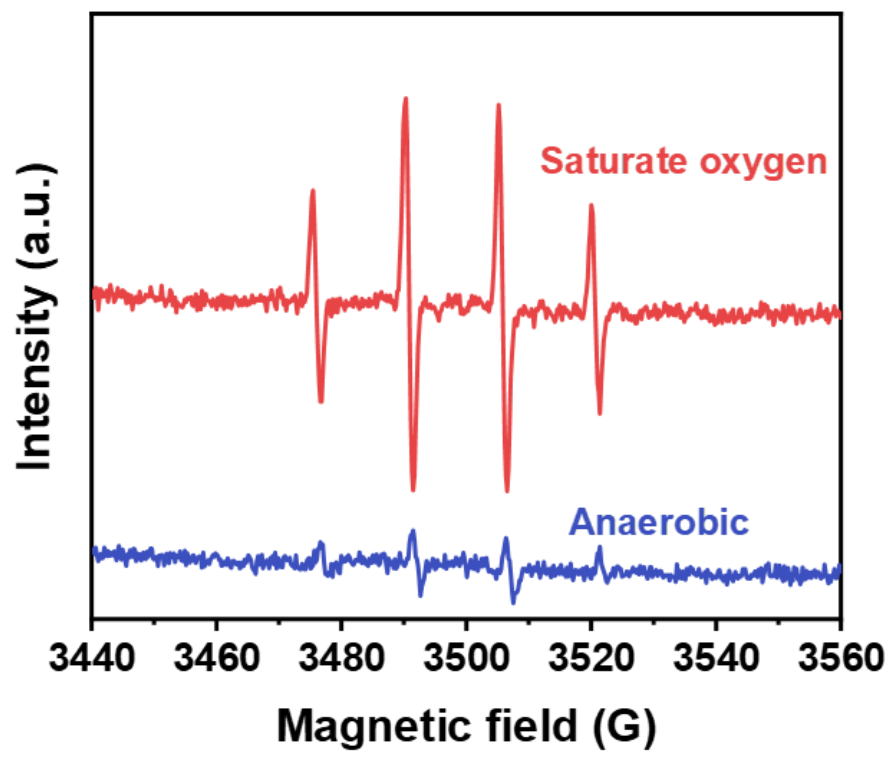

Figure S17. EPR spectra under saturated oxygen or anaerobic conditions of Ag-3/In_2O_3.

In situ EPR spectroscopy under anaerobic conditions, using 5,5–dimethyl–1–pyrroline N–oxide (DMPO) as a spin trap, detected trace amounts of DMPO-·OH radicals adduct formation, which may be attributed to residual traces of oxygen in the system. This indicates that H_2O cannot be converted to ·OH radicals on Ag-3/In_2O_3.

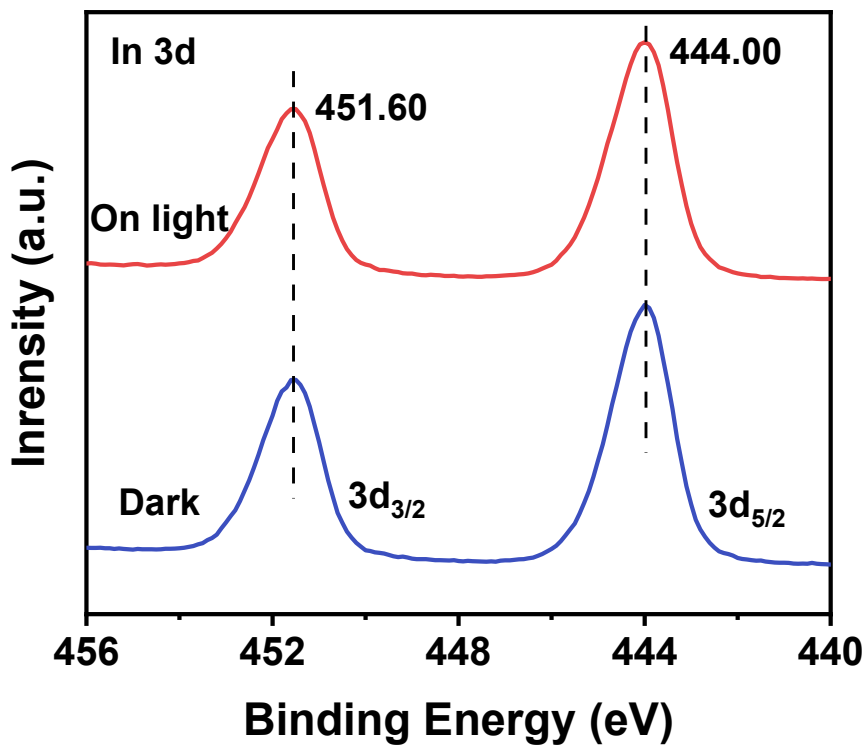

Figure S18. XPS spectra under dark or light conditions of Ag-3/In_2O_3.

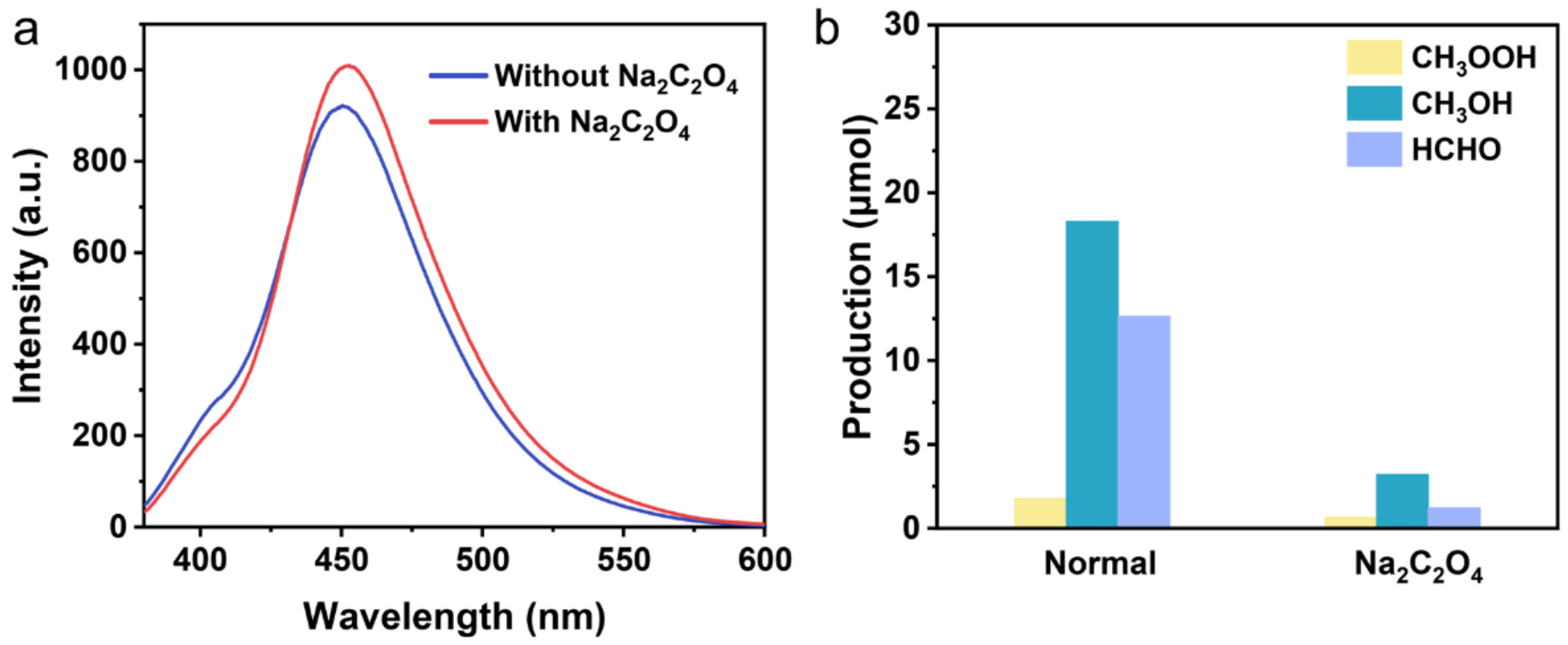

Figure S19. (a) Fluorescence spectra of 7-HC with Ag-3/In_2O_3; (b) Trapping experiments of photogenerated holes.

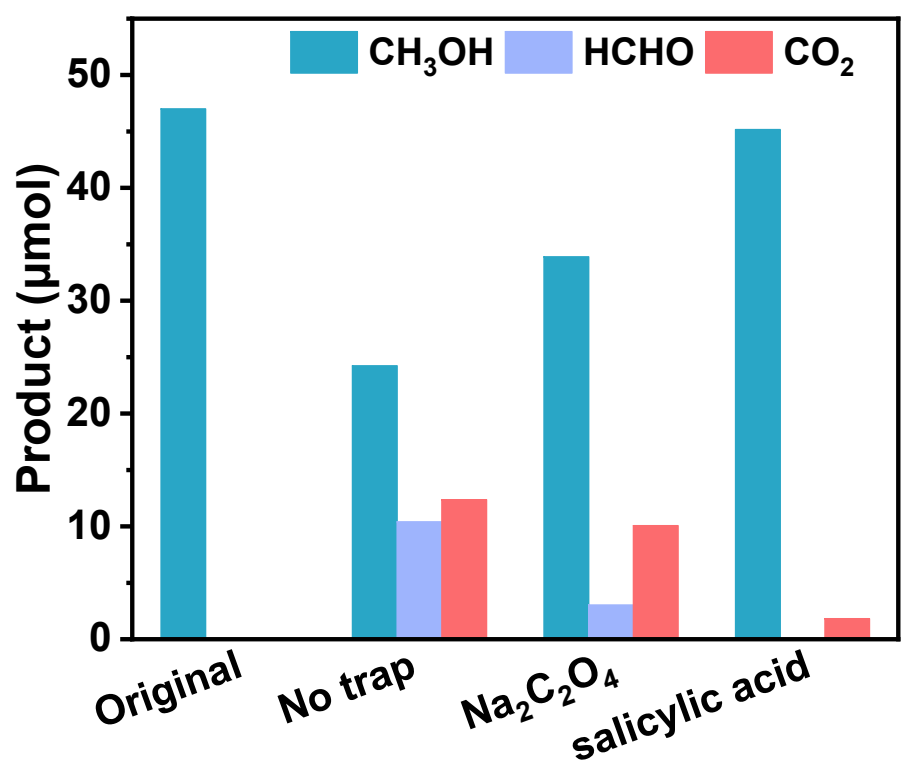

Figure S20. Radical trapping experiments of photo–thermal catalytic CH_3OH oxidation over Ag-3/In_2O_3.

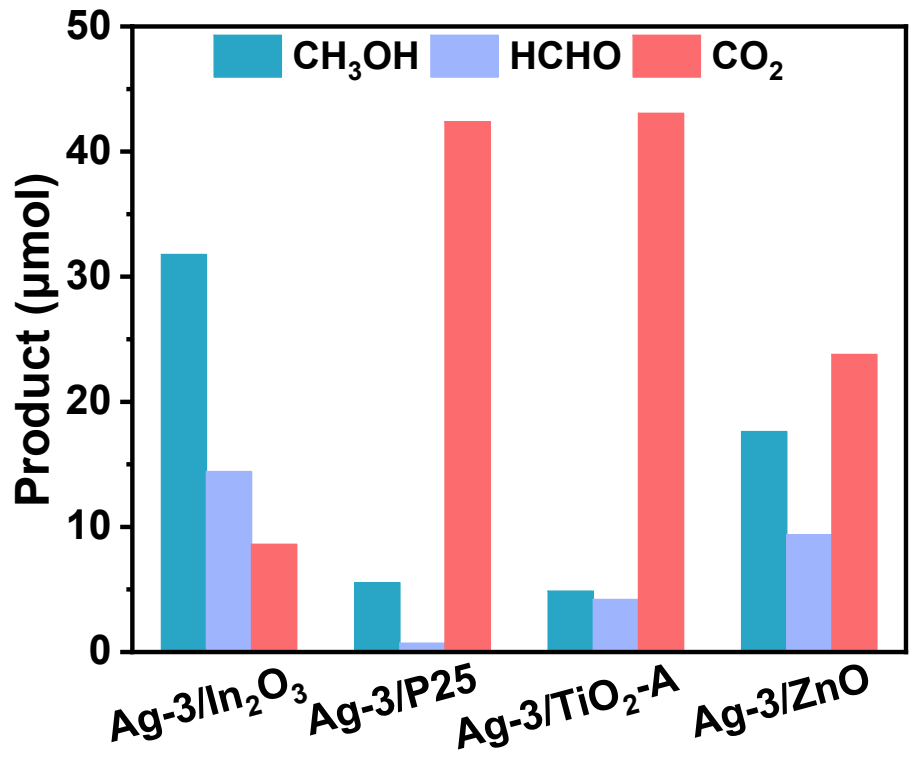

Figure S21. Photo–thermal catalytic CH_3OH oxidation.

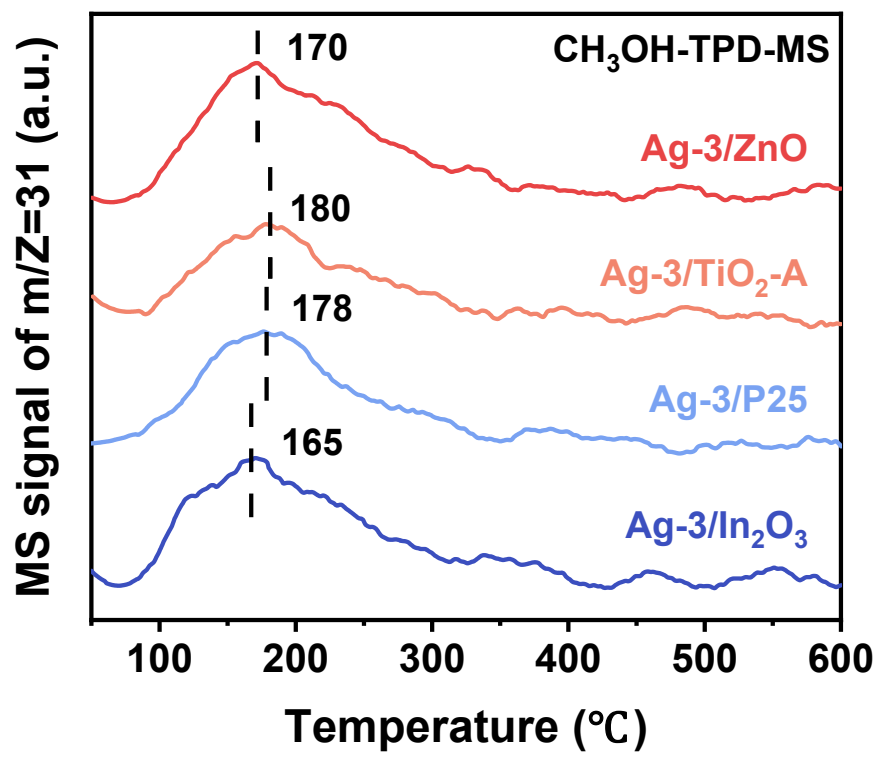

Figure S22. CH_3OH-TPD-MS profiles of different catalysts.

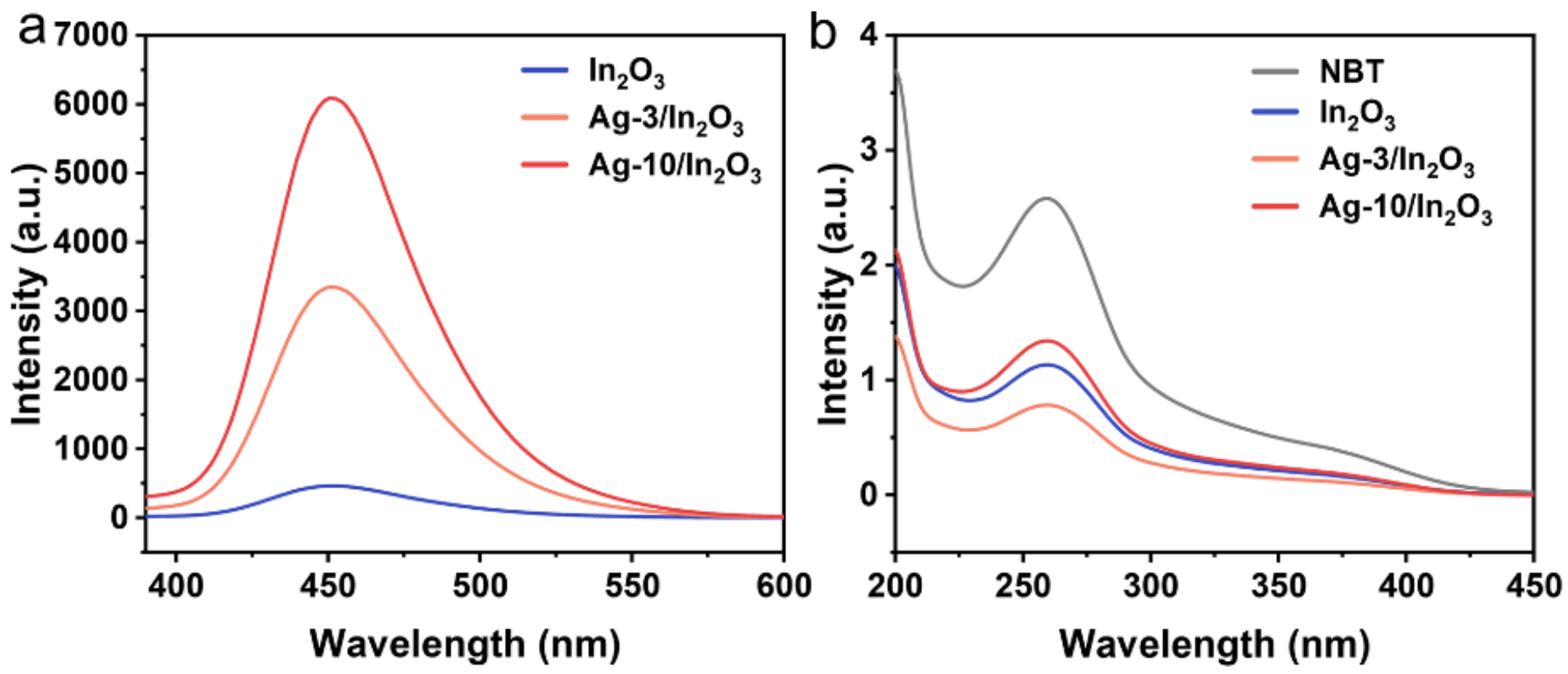

Figure S23. (a) PL spectra of 7-HC induced by ·OH radicals; (b) photodegradation of NBT induced by ·OOH radicals.

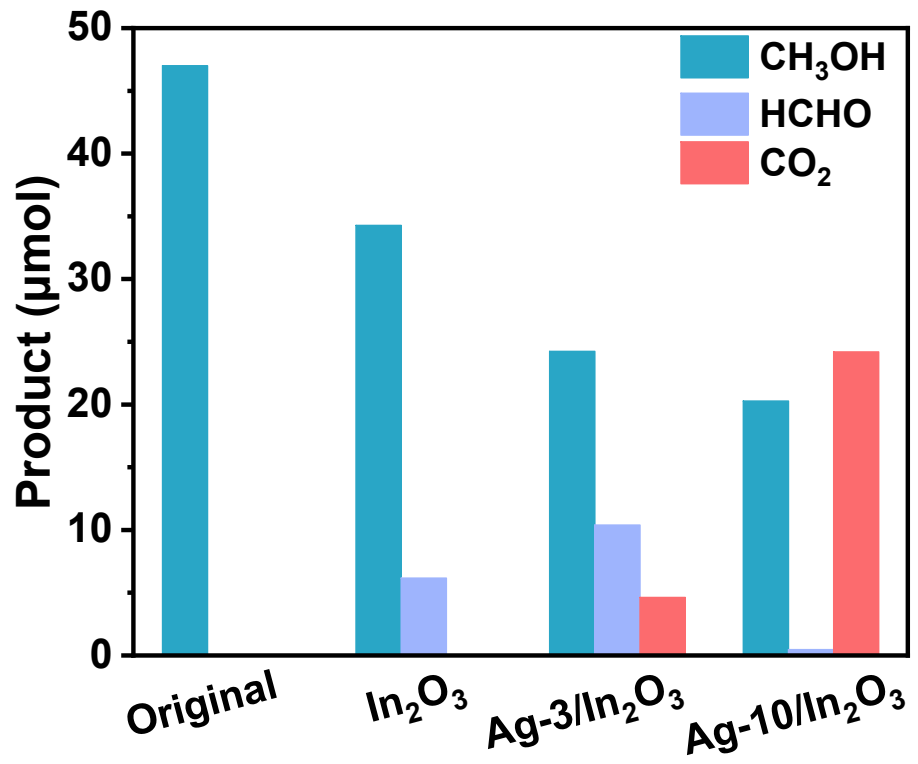

Figure S24. Photo–thermal catalytic oxidation of CH_3OH.

Supporting Table S1. ICP–OES results of catalysts.

Samples	Theory loading (wt.%)	Actual loading (wt.%)
Ag-0.1/In_2O_3	0.1	0.098
Ag-1/In_2O_3	1	0.84
Ag-3/In_2O_3	3	2.91
Ag-10/In_2O_3	10	9.52

Supporting Table S2. Yield and selectivity of liquid oxygenates with varying Ag mass fractions.

catalyst	Yield (μmol)					Liquid oxygenates selectivity (%)
	CH_3OOH	CH_3OH	HCHO	CO_2	Liquid oxygenates	
In_2O_3	0.80	0.80	4.17	0.00	5.77	100
Ag-0.1/In_2O_3	2.30	6.51	9.83	0.00	18.64	100
Ag-1/In_2O_3	0.84	14.16	11.94	0.00	26.94	100
Ag-3/In_2O_3	1.78	18.30	12.64	0.00	32.72	100
Ag-10/In_2O_3	0.09	23.08	2.78	5.47	25.95	82

Supporting Table S3 Reaction condition, production, space–time yield (STY), and selectivity of liquid oxygenates compared with the state-of-the-art works on photocatalytic CH_4 oxidation.

entry	catalyst	Reaction condition	STY ($\mu mol \cdot g^{-1} \cdot h^{-1}$)	Selectivity (%)	Ref.
1	Ag-3/In_2O_3	Xenon lamp (320–780 nm), 5 mg catalyst, 39 bar CH_4, 1 bar O_2, 150ºC, 1 h, 20 mL H_2O	6544	100	This work
2	Ag-ZnO	10 mg catalyst, 1.9 MPa CH_4, 0.1 MPa O_2, 75 mL H_2O, 2 h, 300 W Xe lamp with full-spectrum light source	4060	95	[12]
3	Cu-def-WO_3	5 mg catalyst, 120 mL H_2O, 420 nm light irradiation, 25ºC	2489.5	100	[13]
4	Au_{NPs}/In_2O_3	10 mg catalyst, 10 bar O_2, 20 bar CH_4, 180 mL H_2O, 3 h, Xe lamp (300–1100 nm)	2067	91	[2]
5	$BiVO_4$	10 mg catalyst, 10 bar O_2, 10 bar CH_4, 10 mL H_2O, 3 h, Hg lamp	4370	87	[14]
6	Au BP	200 mg catalyst, 30 bar CH_4, 3 bar O_2,	57	99	[15]

		20 mL H_2O, 90°C, 2 h			
7	0.1 wt.% Ag TiO_2	10 mg catalyst, 2 MPa CH_4, 0.1 MPa O_2, 100 mL H_2O, 25ºC, 2 h, light source: 300 W Xe lamp, 300 < λ < 500 nm	2260	77	[16]
8	Ag/$TiO_{2(001)}$	10 mg catalyst, 100 mL H_2O, 2 MPa CH_4, 0.1 MPa O_2, 25°C, 300 W Xe lamp	5410	90	[17]
9	Au-CoO_x/TiO_2	10 mg catalyst, 2.0 MPa CH_4, 0.1 MPa O_2, 25ºC, 2 h, 100 mL H_2O	2540	60	[18]
10	ZnO (Zni) - 350	1.9 MPa CH_4, 0.2 MPa O_2 2 h, 30℃	3041	99	[19]
11	Pd/H-TiO_2	10 mg cat., 60 mL H_2O, 2 MPa, CH_4:O_2=50:1, 45ºC, 300 W Xe lamp	3070	70	[20]
12	Pd-def-In_2O_3	20 mg catalyst, 50 mL distilled H_2O, 19 bar CH_4, 1bar O_2, 3 h	5000	83	[1]
13	TiO_2@SiO_2-AuPd	10 mg catalyst,100 mL H_2O, 6.9 bar of CH_4, 2.76 bar of O_2, 1 h, 25°C, 365 nm UV LED	5146	94	[21]

14	$Au_{1.0}Fe_{0.33}$-ZnO	40 mg catalyst, 20 mL H_2O, 2.0 bar O_2, 18 bar CH_4, 20ºC, 1 h, stirring rate 500 rpm, 300 W Xe lamp irradiation	1500	95	[22]
15	Pt_{NPs}-WO_3	10 mg catalyst, 19 bar CH_4, 1 bar O_2, 120 mL H_2O, 4 h, 25°C, 365 nm LED (60 $mWcm^{-2}$)	3125	90	[23]
16	Pt_1/ZnO	300 W Xe lamp (320–2500 nm), 10 mg catalyst, 1.9 MPa CH_4, 0.1 MPa O_2, 4 h, 75 mL H_2O	4620	95	[24]
17	Fe−Au/STO_v	20 mg catalyst, 30 mL H_2O, CH_4/O_2=19:1 (bar), 25°C	7600	96	[25]
18	Pd-ZnO	0.1 MPa air, 1.9 MPa CH_4, 1 mg catalyst, 30 mL H_2O, 1 h, 300 W Xe lamp	7000	100	[26]
19	Au/ZnO	2.0 MPa CH_4, 0.1 MPa O_2, 1 h, 10 mg catalyst, 150 mL H_2O	9500	94	[27]
20	$Pd_{0.05}/TiO_2$-800	10 mg catalyst, 2MPa CH_4, 0.1MPa O_2, 25°C, 3 h, 300W Xe lamp	8140	91.6	[28]

21	Cu/ZnO-P	10 mg catalyst, 19 bar CH_4, 1bar O_2,75 mL H_2O, 1 h, 25℃, 300 W Xe lamp	4724.2	99.6	[29]
22	Au/$CuSeO_{2.82}$	20 mg catalyst, 40 mL H_2O, 2 h, 25℃	2300	96	[30]
23	0.5Ru-ZnO	10 mg catalyst, 180 mL H_2O, 20 bar CH_4, 0.75 bar O_2, 150℃, 1 h, 365 nm LED	40150	91	[31]

References

1. Luo L, Fu L, Liu H *et a*l. Synergy of Pd atoms and oxygen vacancies on In_2O_3 for methane conversion under visible light. *Nat Commun* 2022; **13**: 2930.
2. Jiang Y, Li S, Wang S *et al*. Enabling specific photocatalytic methane oxidation by controlling free radical type. *J Am Chem Soc* 2023; **145**: 2698–707.
3. Zhou Q, Tan X, Wang X *et al*. Selective photocatalytic oxidation of methane to methanol by constructing a rapid O_2 conversion pathway over Au–Pd/ZnO. *ACS Catal* 2024; **14**: 955–64.
4. Kresse G and Furthmüller J. Efficient iterative schemes for ab initio total-energy calculations using a plane-wave basis set. *Phys Rev B* 1996; **54**: 11169–86.
5. Kresse G and Joubert D. From ultrasoft pseudopotentials to the projector augmented-wave method. *Phys Rev B* 1999; **59**: 1758–75.
6. Perdew J P, Burke K, and Ernzerhof M. Generalized Gradient Approximation Made Simple. *Phys Rev Lett* 1996; **77**: 3865–8.
7. Blöchl P E. Projector augmented-wave method. *Phys Rev B* 1994; **50**: 17953–79.
8. Grimme S, Antony J, Ehrlich S *et al*. A consistent and accurate ab initio parametrization of density functional dispersion correction (DFT-D) for the 94 elements H-Pu. *J Chem Phys* 2010; **132 (15)**: 154104.
9. Monkhorst H J and Pack J D. Special points for Brillouin-zone integrations. *Phys Rev B* 1976; **13**: 5188–92.
10. Delley B. The conductor-like screening model for polymers and surfaces. *Mol Simul* 2006; **32**: 117–23.
11. Luo G, Jing Y, and Li Y. Rational design of dual-metal-site catalysts for electroreduction of carbon dioxide. *J Mater Chem A* 2020; **8**: 15809–15.
12. Hao Y, Zhao Y, Zhang C *et al*. ZIF-8 derived Ag/ZnO photocatalyst with enriched Ag-ZnO interface for effective oxidation of methane to liquid oxygenates with simultaneous mmol-scale productivity and ~100 % selectivity. *Appl Catal B* 2024; **359**: 124505.
13. Luo L, Han X, Wang K *et al*. Nearly 100% selective and visible-light-driven

methane conversion to formaldehyde via. Single-atom Cu and $W^{\delta+}$. *Nat Commun* 2023; **14**: 2690.

14. Fan Y, Zhou W, Qiu X *et al*. Selective photocatalytic oxidation of methane by quantum-sized bismuth vanadate. *Nat Sustain* 2021; **4**: 509–15.

15. Luo L, Luo J, Li H *et al*. Water enables mild oxidation of methane to methanol on gold single-atom catalysts. *Nat Commun* 2021; **12**: 1218.

16. Song H, Meng X, Wang S *et al*. Direct and selective photocatalytic oxidation of CH_4 to oxygenates with O_2 on cocatalysts/ZnO at room temperature in water. *J Am Chem Soci* 2019; **141**: 20507–15.

17. Feng N, Lin H, Song H *et al*. Efficient and selective photocatalytic CH_4 conversion to CH_3OH with O_2 by controlling overoxidation on TiO_2. *Nat Commun* 2021; **12**: 4652.

18. Song H, Meng X, Wang S *et al*. Selective photo-oxidation of methane to methanol with oxygen over dual-cocatalyst-modified titanium dioxide. *ACS Catal* 2020; **10**: 14318–26.

19. Xiao Z, Wan Z, Zhang J *et al*. Interstitial zinc defects enriched ZnO tuning O_2 adsorption and conversion pathway for superior photocatalytic CH_4 oxygenation. *ACS Catal* 2024; **14**: 9104–14.

20. Zhang X, Wang Y, Chang K *et al*. Constructing hollow porous Pd/H-TiO_2 photocatalyst for highly selective photocatalytic oxidation of methane to methanol with O_2. *Appl Catal B* 2023; **320**: 121961.

21. Xie C, Sun E, Wan G *et al*. Transport mediating core-shell photocatalyst architecture for selective alkane oxidation. *Nano Lett* 2023; **23**: 2039–45.

22. Du H, Li X, Cao Z *et al*. Photocatalytic O_2 oxidation of CH_4 to CH_3OH on AuFe-ZnO bifunctional catalyst. *Appl Catal B* 2023; **324**: 122291.

23. Zhai G, Yang S, Chen Y *et al*. Direct photocatalytic oxidation of methane to formic acid with high selectivity via a concerted proton-electron transfer process. *J Am Chem Soci* 2025; **147**: 2444–54.

24. Jiang J, Li Z, Liu W *et al*. Methyl radical dominated highly selective methane oxidation to liquid oxygenates. *J Am Chem Soci* 2025; **147**: 36016–26.

25. Zhu Y, Li Z, Bian J *et al*. Highly selective photo-oxidation of methane to methanol by Fe–Au site-supported $SrTiO_3$ hollow nanotubes with oxygen vacancies. *J Am Chem Soci* 2025; **147**: 34959–71.

26. Peng H, Xue F, Ji Y *et al*. Continuous flow photosynthesis of methanol from methane by plasmonic charge accumulation. *Nat Commun* 2025; **17**:344.

27. Xiao Z, Liang S, Shen J *et al*. Steering products selectivity by intermediate adsorption site as reaction switch for photocatalytic CH_4 conversion. *Appl Catal B* 2026; **384**: 126195.

28. Sun K, Pei X, Wang S *et al*. Synergistic enhancement of electron and hole transport by Pd single atoms and gradient-distributed Ti^{3+} species for high-performance photocatalytic oxidation of CH_4 to oxygenates. *Angew Chem In Ed* 2025; **64**: e202517585.

29. Liu D, Chen F, He Y *et al*. Intensifying internal electric field on ZnO nanoplates with polar surface exposure and Cu doping for efficient photocatalytic methane oxidation to oxygenates. *Adv Funct Mater* 2026; **36 (18)**: e20271.

30. Zhang X, Jiang Y, Gu H *et al*. Near-infrared photon-triggered CH_4-to-CH_3OH conversion over plasmonic oxyselenides. *Nat Commun* 2025; **16**: 7612.

31. Xu Y, Wang C, Li X *et al*. Efficient methane oxidation to formaldehyde via photon–phonon cascade catalysis. *Nat Sustain* 2024; **7**: 1171–81.
